# Single-cell and spatial dissection of necroptosis spatiotemporal evolution driving lymph node metastasis in gastric cancer

**DOI:** 10.1038/s41420-025-02815-z

**Published:** 2025-11-17

**Authors:** Yuhua Hu, Feng Shen, Honghong Zhang, Zhuowen Long, Xiaojun Zhao, Jiale Wen, Yijian Sheng, Junxing Huang, Yan Chen, Qing Guo

**Affiliations:** 1 Graduate school, China Medical University, Shenyang, Liaoning China; 2 The Affiliated Taizhou People’s Hospital of Nanjing Medical University, Taizhou School of Clinical Medicine, Nanjing Medical University, Taizhou, Jiangsu China; 3 Nanjing University of Chinese Medicine, Nanjing, China; 4 The Affiliated PanJin Central Hospital, PanJin, Liaoning China; 5 Institution, Lianchuan Biotechnology Corporation Limited, Hangzhou, China

**Keywords:** Cancer microenvironment, Metastasis, Cancer genomics

## Abstract

Lymph node metastasis is a common metastatic route of gastric cancer. However, the heterogeneity of tumor cells and tumor microenvironment between primary gastric cancer and metastatic lymph nodes, as well as the driving mechanisms of metastasis, remain not fully understood. In this study, single-cell RNA sequencing and spatial transcriptomics analysis were performed on 4 gastric cancer patients (four primary tumors and two paired metastatic lymph nodes). Additionally, the TCGA database was used as an external validation dataset to validate the unfavorable clinical outcomes of necroptosis genes and the correlation between MDK-NCL and immune infiltration. Our single-cell analysis revealed significant heterogeneity in the tumor microenvironment between primary and metastatic lymph nodes of gastric cancer. Pseudotime analysis indicated that gastric cancer cells may follow two potential differentiation trajectories during lymph node metastasis. One type of cells possess the ability to migrate to lymph nodes, while the other type remain in the primary site during tumor progression. We observed that the expression of necroptosis-related gene CHMP3 was significantly associated with lymph node metastasis. Immunofluorescence further suggested upregulated CHMP3 protein expression in metastatic lymph node. Furthermore, we found that MDK-NCL interaction was active in metastatic lymph nodes. External validation using the TCGA cohort indicated that high MDK-NCL expression correlated with an immunosuppressive microenvironment. Finally, through integrated single-cell and spatial analysis, we observed that gastric cancer cells may remodel the tumor microenvironment via the MDK-NCL signaling pathway. In summary, our study revealed the dynamics of tumor cells in lymph node metastasis in GC and identified a subtype of GC cells with potential metastatic capability. Our data suggest that necroptosis and the MDK-NCL signaling pathway may be involved in facilitating lymph node metastasis, providing new insights into the mechanisms of GC progression and potential therapeutic targets.

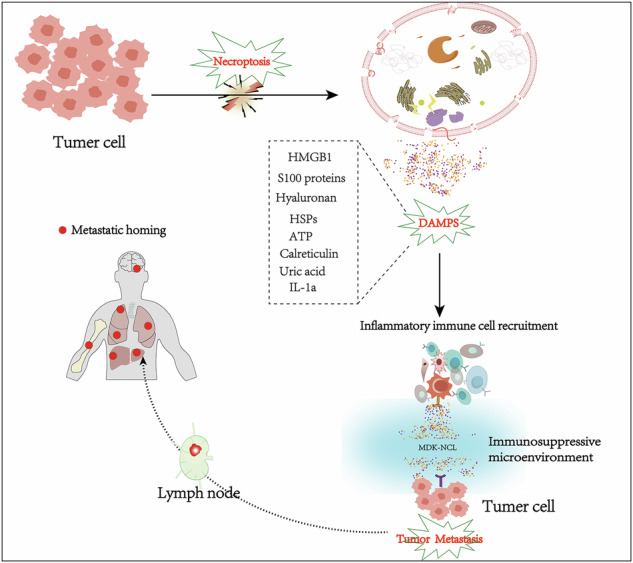

## Background

Gastric cancer (GC) is one of the top five deadliest cancers worldwide and ranks as the third leading cause of cancer-related deaths [1]. Despite advances in surgical treatment, the overall 5-year survival rate for GC patients remains below 30% [2], due largely to late diagnosis and frequent lymph node metastasis (LNM)—the most common route of GC dissemination [3]. Patients with lymph node metastasis experience significantly shorter overall survival compared to those without [4]. Although systemic therapies for GC—including chemotherapy, targeted agents, and immunotherapy—have improved in recent years, the prognosis for advanced-stage patients remains poor. Crucially, the heterogeneity of GC tumor cells and the tumor microenvironment (TME), as well as the mechanisms underlying LNM, are still incompletely characterized.

Increasing evidence suggests that the heterogeneity of the TME and tumor cells plays a crucial role in tumor progression and metastasis [5–7]. Single-cell RNA sequencing (scRNA-seq) has revolutionized our understanding of cellular diversity in malignancies, including GC [8], enabling insights into malignant cell differentiation [9], immune cell functional states [10], and stromal reprogramming [11]. However, a key limitation of scRNA-seq is the loss of native spatial architecture due to tissue dissociation [12]. This shortcoming is now addressed by spatial transcriptomics (ST) [13, 14], and the integration of scRNA-seq with ST allows high-resolution mapping of cellular ecosystems while preserving spatial context [15], offering unprecedented opportunities to decode metastasis.

In this study, we applied integrated scRNA-seq and ST to profile four primary gastric tumors and two paired metastatic lymph nodes. Through trajectory analysis, we reconstructed the metastatic progression of GC cells to lymph nodes and identified two distinct differentiation directions with divergent malignant potential. We further uncovered a subset of gastric cancer cells exhibiting enhanced potential for lymph node metastasis. Our data suggest that necroptosis may contribute to GC lymph node metastasis, and we found that MDK–NCL signaling—a pathway linked to metastasis—was activated in metastatic lymph nodes. Spatial transcriptomic localisation revealed enrichment of MDK–NCL at the tumor–stroma interface, suggesting a role in mediating immunosuppression. Collectively, our work leverages integrated single-cell and spatial transcriptomic profiling to identify a metastasis-prone GC cell subset and trajectory, and further uncovered necroptosis as a key mechanistic driver of lymph node metastasis, mediated through DAMP release and activation of the MDK-NCL signaling axis at the tumor-stroma interface. Our findings provide new insights into the mechanisms of GC metastasis and highlight potential therapeutic strategies targeting necroptosis-associated immunosuppression.

## Results

### Single-cell transcriptome profiling of gastric cancer: comparative analysis of primary tumors and metastatic lymph nodes

**Fig. 1:**
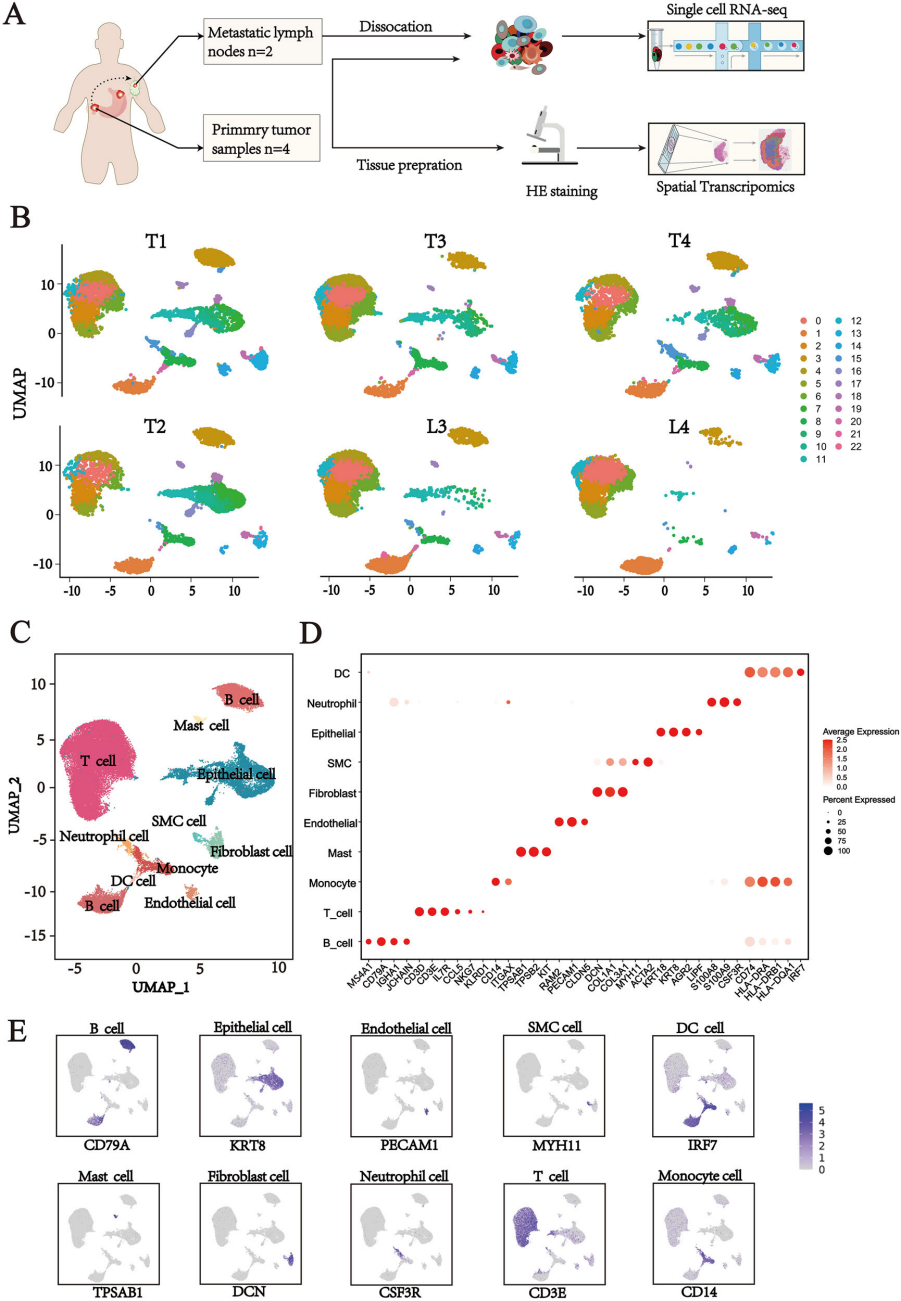
Single-cell transcriptomic analysis of the cellular composition in matched primary gastric tumors and metastatic lymph nodes. **A** Schematic diagram of the experimental workflow. We collected four primary tumor tissue samples (PT, *n* = 4) and two metastatic lymph node samples (MLN, *n* = 2) from four gastric cancer patients at Taizhou People’s Hospital affiliated with Nanjing Medical University. These samples were subjected to scRNA-seq and ST analysis using the 10x Genomics platform. **B** Single-cell UMAP plots for the six samples, with each plot displaying 23 cell clusters. Different colors represent different cell populations. **C** UMAP plot of 68 838 cells profiled in the present study colored by major cell types. **D** Bubble plots of the marker genes expressed in the major cell types. Dot color reflects expression level and dot size represents the percent of cells expressing marker genes in different cell types. **E** Expression of characteristic marker genes for the major cell types.

To comprehensively characterize the cellular composition and spatial architecture of PT and MLN, we performed scRNA-seq and spatial ST analysis on four GC tissue samples and two paired MLN samples obtained from patients at Taizhou People’s Hospital Affiliated to Nanjing Medical University using the 10x Genomics platform (Fig. 1A). After stringent quality control filtering for genes and cells (see Methods Section), we constructed a cellular atlas comprising 68,838 high-quality single cells, with a median of 1,340 genes detected per cell. Integration of transcriptional profiles across all samples revealed 22 distinct cell clusters, visualized in a Uniform Manifold Approximation and Projection (UMAP) (Fig. 1B and Figure S1A, C, Supporting Information). We further generated a UMAP representation to illustrate sample-wise distribution and employed canonical marker genes to annotate major cell types (Fig. 1C).

Based on established marker expression, we identified the following cell populations: B cells (CD79B, CD79A, MS4A1), endothelial cells (VWF, CDH5, PECAM1), epithelial cells (CDH1, KRT8, EPCAM), fibroblasts (PDGFRB, COL1A2, DCN), mast cells (SLC18A2, FCER1A, TPSB1), T cells (TRBC2, CD2, CD3E), monocytes (CD14, ITGAX), neutrophils (CSF3R, S100A8, S100A9), smooth muscle cells (MYH11, ACTA2), and dendritic cells (IRF7, HLA-DRA, HLA-DRB1) [16] (Fig. 1D). For each cell type, a representative marker gene was selected and confirmed to exhibit specific expression across corresponding clusters (Fig. 1E and Fig. S1B, Supporting Information).

### Expression changes of gastric cancer cells in primary tumors and lymph node metastases

**Fig. 2:**
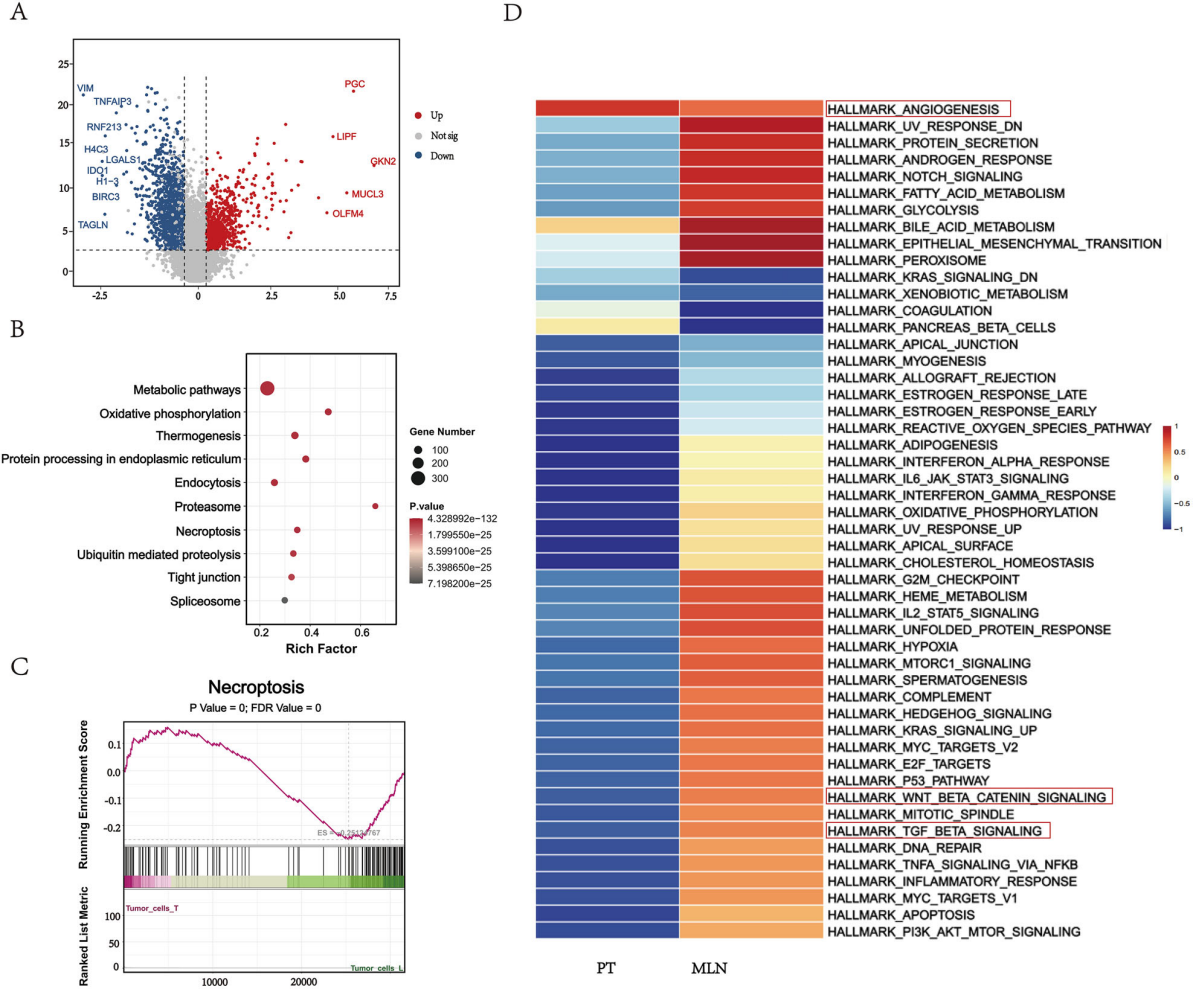
Differential expression patterns of gastric cancer cells in PT and MLN. **A** Volcano plot showing differentially expressed genes (DEGs) detected in PT and MLN. Genes down-regulated in MLN are shown in blue and up-regulated genes are shown in red. **B** Bubble plots showing the results of KEGG enrichment analysis. The size of the bubbles represents the number of genes involved in the corresponding KEGG term, and the color gradient from blue to red indicates the adjusted *p*-value. **C** GSEA analysis of the necroptosis pathway. **D** Heatmap displaying GSVA scores for the gene signatures associated with critical pathways in PT and MLN.

To further characterize the molecular features of GC cells during LN metastasis, we compared the transcriptomes of GC cells from PT and MLN. This analysis identified 913 downregulated and 347 upregulated genes in MLN-derived GC cells (Fig. 2A). KEGG pathway enrichment analysis of these differentially expressed genes revealed that the top pathways were involved in energy metabolism, protein processing, transport, and gene regulation (Fig. 2B). Notably, the necroptosis pathway was among the most significantly enriched pathways in MLN. Necroptosis is an inflammatory type of programmed cell death that can exert pro-tumor effects by shaping an immunosuppressive microenvironment; its role, however, is highly context-dependent. Previous studies have reported that receptor-interacting protein kinase (RIPK) 1, one of the markers of necroptosis, is highly expressed in GC tissue and positively correlated with the clinical stage of GC and lymph node metastasis. Consistent with their findings, we also observed this form of programmed cell death in our study of gastric cancer. GSEA results for GC cells showed activation of necroptosis in GC cells within MLN (Fig. 2C). This finding suggests that this pathway may contribute to LN metastasis in GC. To gain a broader understanding of pathway-level alterations, we performed gene set variation analysis (GSVA) comparing PT and MLN transcriptional profiles (Fig. 2D). The results confirmed distinct gene expression programs between the two sites. In agreement with the GSEA results, epithelial-mesenchymal transition (EMT) was highly active in MLN, indicating that metastatic gastric cancer cells experience an active EMT process. Notably, the Wnt/β-catenin signaling pathway and TGF-β signaling pathway had higher GSVA scores in MLN, suggesting their association with the metastasis process. However, the angiogenesis pathway had similar GSVA scores in primary tumors and metastatic lymph nodes, which appears to contradict previous reports that newly developed lymph node metastases do not form new blood vessels [17,18]. A possible explanation is that the pathway enrichment reflects survival strategies of tumor cells adapting to the lymph node microenvironment (e.g., vasculogenic mimicry) rather than driving angiogenesis [19].

### The developmental trajectory of gastric cancer cells during lymph node metastasis

**Fig. 3:**
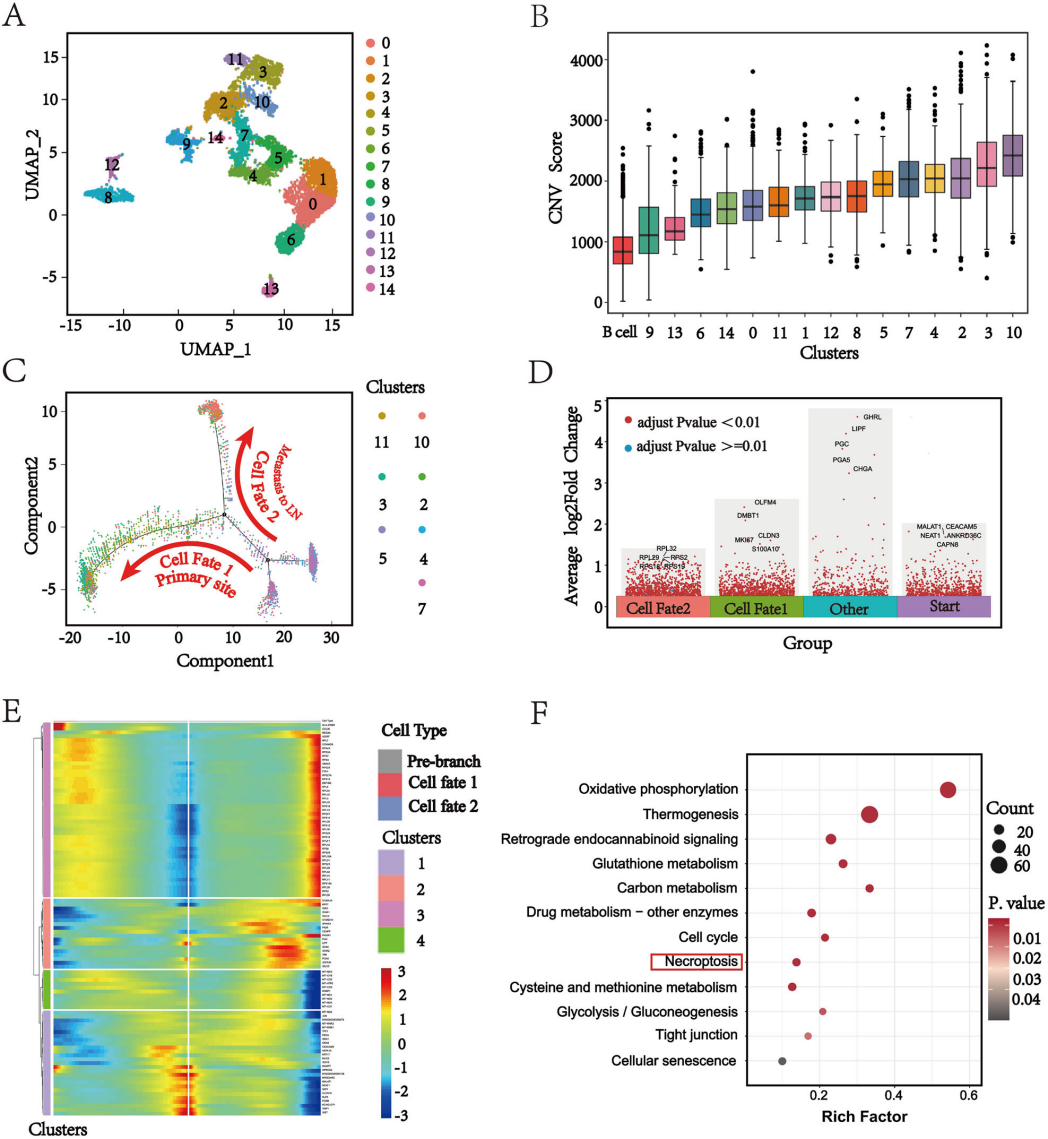
Single-cell analysis of the metastatic mechanism of gastric cancer to lymph nodes. **A** High-resolution t-SNE analysis was performed to recluster epithelial cells, revealing 15 distinct cell clusters. Different colors represent different cell clusters. **B** A violin plot of copy number variation (CNV) levels in different epithelial cell clusters reflects the malignant characteristics of tumor cells. **C** The single-cell trajectory plots generated by Monocle [44] illustrate the reconstructed developmental pathways of tumor cells originating from PT and MLN. The trajectory features two primary branches: one is identified as the pathway of lymph node migration, while the other is deemed the pathway of primary tumor stay. **D** A volcano plot illustrates the differential gene expression across the different trajectories. **E** The heatmap shows the gene expression trend of each branch. The center of the heat map represents the start of the trajectory; moving from the center to the right represents differentiation from the start to cell fate 2 (LN metastasis branch), and moving from the center to the left represents differentiation from the root to cell fate 1 (PT branch). **F** Enrichment analysis results for the GCs clusters in the metastatic branch.

Considering that gastric cancer cells originate from epithelial cells, we performed high-resolution UMAP analysis on all epithelial cells, and reclassified them into 15 clusters (Fig. 3A). We then constructed evolutionary trees for epithelial cells from each patient, all of which showed similar trajectories from PT to MLN, indicating that tumor evolution during gastric cancer dissemination shares roughly similar characteristics (Fig. S2A, B, Supporting Information). Copy number variation (CNV) analysis is widely used in scRNA-seq to differentiate between benign and malignant cells [20]. By CNV analysis, we evaluated the CNV levels of all epithelial cell clusters and combined with the expression of gastric cancer cell markers, we ultimately identified clusters C2, C3, C4, C5, C7, C10, and C11 as tumor cells (Fig. 3B, and Fig. S2C, D, Supporting Information).

Given the distinct expression patterns of GC cells in PT versus MLN, we constructed a differentiation trajectory for the 3,403 identified tumor cells to investigate their transition from primary to metastatic sites. This analysis successfully reconstructed a developmental trajectory illustrating the progression of GC cells from the primary site to metastasis (Fig. 3C).

This trajectory mainly contains a starting point and two branches. Clusters C4, C5, and C7, mainly originating from the PT, were identified as the starting point of this cell differentiation trajectory, due to their lower CNV levels and being located at the root of the trajectory. After identifying the beginning of the trajectory, the differentiation trajectory of GC cells was divided into two branching directions from the starting point towards Cell fate 1 or Cell fate 2. The Cell Fate 1 consisted mainly of PT-derived clusters C2 and C3. In contrast, the Cell Fate 2 consisted mainly of clusters C10 and C11, which displayed higher CNV levels and were predominantly comprised of GC cells from MLN (Fig. S2B, Supporting Information), implying that cells in this branch possess enhanced invasive or metastatic potential. Therefore, we designated the Cell Fate 2 as the “metastatic branch” and the Cell Fate 1 as the “primary branch.”

Based on these findings, we hypothesize that two functionally distinct subtypes of GC cells may coexist within primary tumors: one with an inherent potential to migrate to lymph nodes, and another that is predominantly retained at the primary site during progression. Specifically, clusters C2 and C3 appear to represent a primary-restrained subtype, whereas clusters C10 and C11 constitute a metastasis-prone subtype.

### The potential key regulatory role of necroptosis in gastric cancer lymph node metastasis

To investigate dynamic gene expression changes during metastasis, we performed gene enrichment analysis on the 8,897 genes identified from single-cell trajectory analysis, based on their patterns across different branching trajectories (Fig. 3D, and Fig. S2E, F, Supporting Information). The heatmap illustrates the transition of gene expression from the initial state to cell fate 1 or 2 and reveals four different modes of transformation (Fig. 3E). The metastatic branch (Cell Fate 2) was significantly enriched in classical metastasis-associated pathways, including cell cycle, P53 signaling, cell junctions, mismatch repair, cytoskeleton regulation, and gap junctions (Fig. 3F). These pathways align with the clinicopathological features of the patients and the highly malignant phenotype of the metastatic clusters. Notably, the metastatic branch was also significantly enriched in genes related to the necroptosis pathway, suggesting that this form of regulated cell death may be involved in the lymphatic metastasis of gastric cancer, reflecting the unique biological characteristics of metastatic cells.

The role of necroptosis in tumor progression remains complex and context-dependent. Initially proposed as a backup cell death mechanism to overcome apoptosis resistance in cancer therapy [21], recent evidence suggests that damage-associated molecular patterns (DAMPs) can remodel the TME into an immunosuppressive niche, thereby promoting tumor growth and metastasis [22, 23]. For example, potassium ions released from necrotic cells were found to significantly inhibit CD8 + T cell killing function in melanoma studies [24]. And pancreatic cancer model showed that necroptosis induced immune tolerance TME through the CXCL1-CXCR2 and SAP130-Mincle signaling axis [25].

**Fig. 4:**
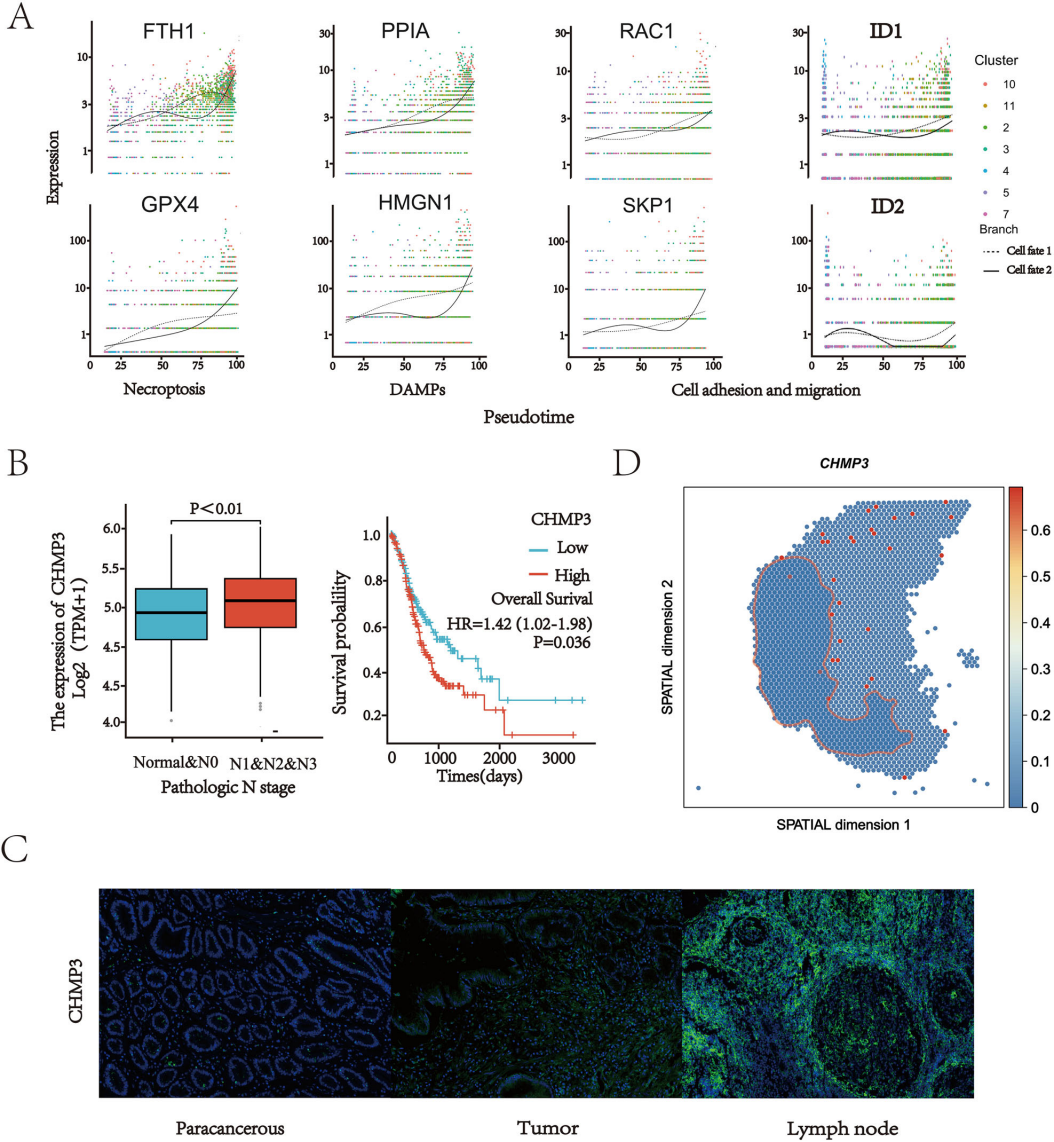
The dynamic expression of related genes in the process of metastasis and the correlation with patient prognosis. **A** Dot plots of dynamic expression of key genes in DAMPs and Necroptosis and the two pathways themselves based on KEGG database along two cell fates. **B** Prognostic and clinicopathologic correlation of necroptosis related genes in gastric adenocarcinoma (TCGA cohort, *n* = 407). Kaplan–Meier curves showed that patients with high necroptosis related genes expression had significantly shorter overall survival. Elevation of necroptosis related genes expression was associated with advanced N stage. **C** Immunofluorescence staining of CHMP3 green in PT and MLN and paracancerous issues. Cell nuclei were counterstained with DAPI (blue). Scale bar: 100 μm. **D** Spatial transcriptomic localization of CHMP3, where the regions marked by red lines are tumor tissues and the rest are tumor stroma.

Consistent with these reports, we observed a gradually increasing expression pattern of necroptosis-related genes (e.g., FTH1, GPX4, CHMP3) and DAMP molecules (e.g., PPIA, HMGN1) along the metastatic trajectory. A similar upregulation trend was noted for genes involved in cell adhesion and migration (CCND1, RAC1, SKP1, ID1, ID2). This coordinated dynamic expression pattern suggests that gastric cancer cells undergo complex molecular reprogramming and phenotypic remodeling during metastasis (Fig. 4A).

Given the observed activation of necroptosis during lymph node metastasis, we sought to evaluate its clinical relevance. Using data from 407 gastric adenocarcinoma patients in the TCGA database, we examined the prognostic impact of necroptosis-related gene CHMP3. Univariate logistic regression analysis indicated that high expression of CHMP3 was significantly associated with lymph node metastasis (OR = 1.934, 95% CI: 1.223–3.058; *P* = 0.005) (Supplementary Table 2). Moreover, high necroptosis-related genes CHMP3 expression correlated with advanced N stage and shorter overall survival (Fig. 4B).

We further validated this association using immunofluorescence staining on paired samples from the same patients, including adjacent normal tissues, primary tumor, and metastatic lymph nodes. Necroptosis-related protein CHMP3 expression exhibited a gradual increase from normal tissue to primary tumor and further to metastatic lymph nodes (Fig. 4C). Spatial transcriptomic analysis also revealed specific enrichment of CHMP3 at the tumor–immune interface, implying a potential role in immune cell recruitment or polarization (Fig. 4D). Although these findings highlight a strong association between necroptosis-related protein CHMP3 and metastasis, they indicate correlation rather than causation. Further functional studies are essential to establish a causal relationship.

In conclusion, our study suggests that during gastric cancer metastasis, a subset of cancer cells may release DAMPs via necroptosis, potentially facilitating metastatic progression. This implies that necroptosis may play a key role in lymph node metastasis of gastric cancer.

### Differences in intercellular interaction analysis between PT and MLN

**Fig. 5:**
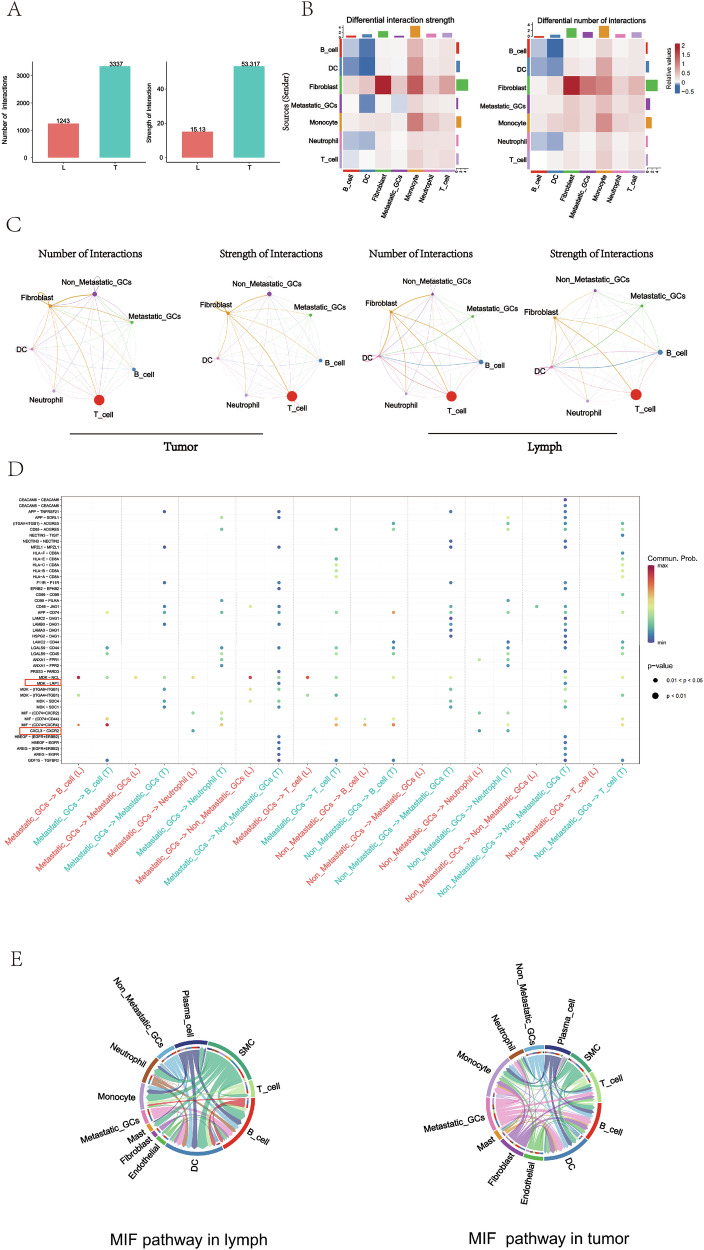
Intercellular networks in PT and MLN revealed by CellChat. **A** The bar chart illustrates the number and strength of intercellular interactions in the MLN and PT. The heat maps show the differences in the intensity (**B**) and quantity (**C**) of intercellular interactions between gastric cancer tumors and lymph nodes. **D** Bubble plots illustrate the significantly differentially expressed ligand-receptor pairs between lymph nodes and tumors. The color of each dot indicates the communication probability, while the size of the dot represents the computed *p*-value. Different cell groups are depicted in distinct colors. Empty spaces signify a communication probability of zero. *p*-values are computed from a two-sided permutation test. **E** The chord diagram depicts the inferred intercellular communication network of MIF signaling in metastatic lymph nodes and tumor tissues. Arc length represents the number of cells in each cell group and edge width represents the communication probability.

To explore the potential interactions among GC cells and other types of cells in the TME of PT and MLN, we mapped intercellular signaling networks using CellChat analysis [26]. The results revealed that both the number and strength of interactions were greater in PT than in MLN, with T cells dominating the communication networks in both tissues (Fig. 5A–C). Further analysis of specific ligand-receptor interactions identified significant upregulation of MIF-(CD74 + CXCR4) signaling in both PT and MLN. This was characterized by high expression of macrophage migration inhibitory factor (MIF) in cancer cells, which signaled to B cells expressing the CD74 and CXCR4 receptors (Fig. 5D).

Interestingly, we found that the ligand-receptor pair MDK - NCL between tumor cells and immune cells was significantly up-regulated in MLN (Fig. 5D), suggesting that this pathway may play an important role in tumor immune responses. It has been shown in endometrial cancer [27], esophageal cancer [28], liver cancer [29], and ovarian cancer [30] that the MDK - NCL signaling pathway affects the tumor immune environment through a variety of mechanisms, including promotion of immunosuppression, reduction of anti-tumor activity of immune cells, promotion of immune escape of tumor cells, and remodeling of tumor microenvironmental cytokine network, which ultimately favors tumor growth and proliferation.

### Immune suppressive environment induced by the MDK-NCL signaling

To evaluate the potential role of the MDK-NCL signaling axis in lymph node metastasis of gastric cancer, we first analyzed its expression patterns using pan-cancer data from the TCGA library. Both MDK and NCL exhibited significantly high expression across multiple cancer types, with their expression levels in GC tissues notably higher than in normal gastric tissues (Fig. S3A, Supporting Information).

**Fig. 6:**
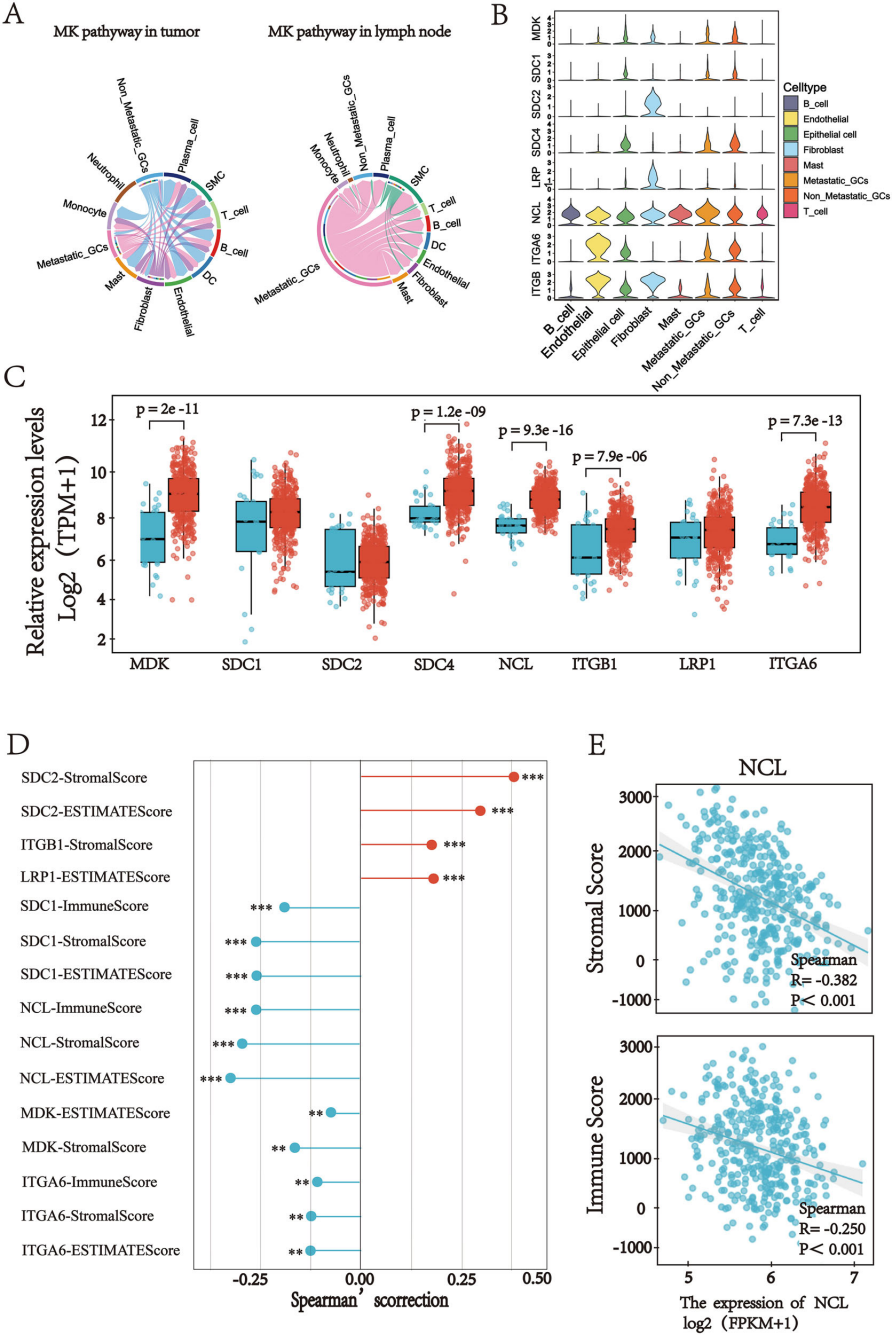
Expression of the Eight Genes Involved in the MK Signaling Network. **A** The chord diagram illustrates the inferred intercellular communication network of the MK signaling pathway. **B** The violin plot displays the expression levels of the eight genes participating in the MK signaling network. **C** The box plot shows the expression levels of the eight genes in 375 gastric adenocarcinoma (GC) tissues and 32 normal gastric tissues. **D** The lollipop chart highlights the significant correlations between the eight genes and the scores estimated using the “estimate” package from TCGA data. **E** The scatter plot demonstrates the Spearman correlation between NCL and ImmuneScore or StromalScore.

As mentioned previously, the MDK-NCL signaling is active between GC cells and immune cells, and in particular in MLN (Fig. 6A). This implied that the MDK-NCL signaling plays a key role in MLN and may contribute to the spread of gastric cancer (Fig. 6A). Figure 6B shows the expression levels of ligands and receptors in the MDK-NCL signaling, in which midkine (MK, MDK) was mainly expressed in epithelial cells, metastatic tumor cells, and fibroblasts, and nucleolin (NCL) was expressed in all eight cell types.

In our cohort, the expression levels of MDK and NCL in GC tissues were higher than those in normal tissues (Fig. 6C). To investigate the functional impact of this axis, we calculated ImmuneScore, StromalScore, and ESTIMATEScore using the ESTIMATE package on the TCGA cohort and assessed their correlation with genes in the MDK-NCL signaling (Fig. 6D). Most genes, especially NCL and SDC1, showed significant inverse correlations with ImmuneScore, StromalScore, and ESTIMATEScore (Fig. 6E, and Fig. S3B, C, Supporting Information), suggesting a strong association between enhanced MDK-NCL signaling and an immunosuppressive microenvironment.

Based on these consistent correlations, we propose a model wherein GC cells may potentially suppress immune cell responses within the TME via the MDK-NCL signaling axis.

### Spatial transcriptomics combined with scRNA-Seq reveals spatial characteristics of gastric cancer metastasis

**Fig. 7:**
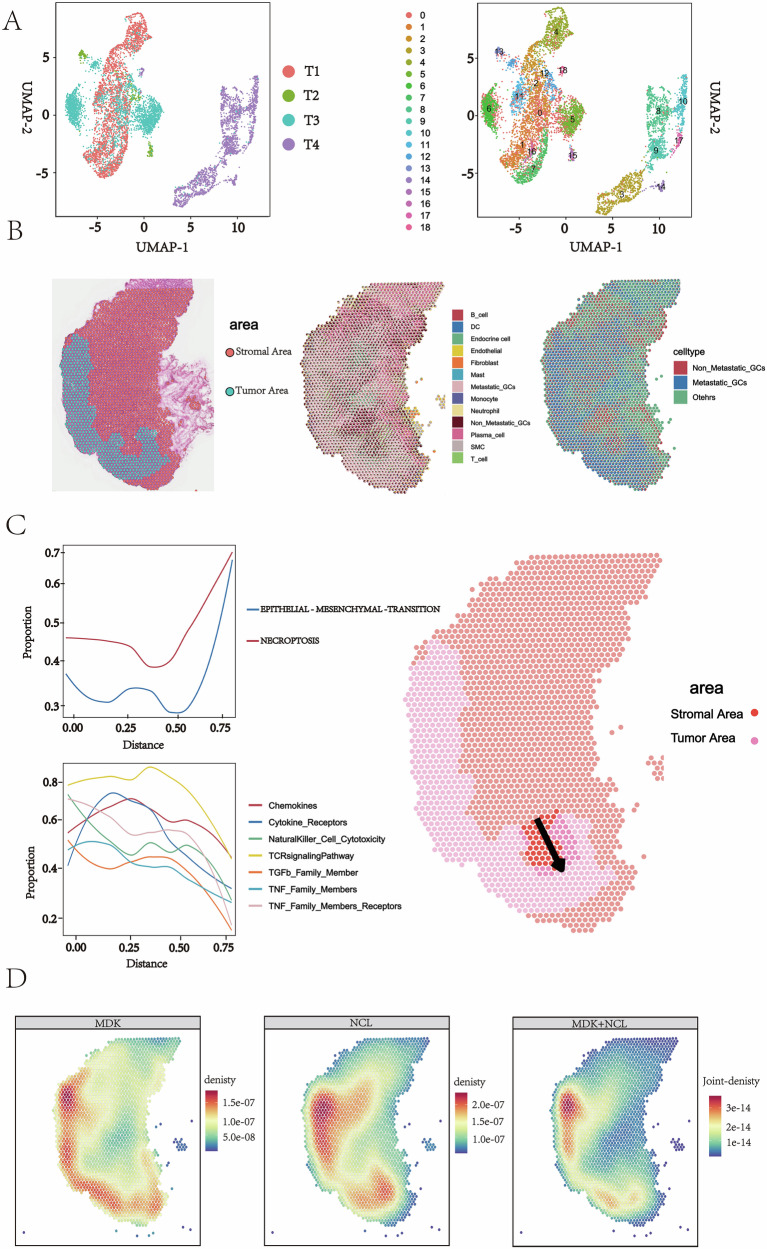
Spatial and molecular dynamics of gastric cancer metastasis revealed by integrated ST. **A** UMAP plots from four primary gastric cancer ST data. Each cluster is shown in a different color. **B** Spatial annotation of tumor and stromal regions. Left: H&E staining of a gastric cancer section from patient T1. The blue part is the tumor region. Middle: The pie chart shows the composition of cell type occupancy for each spatial SPOT (scale bar: 500 μm). Right: proportions of non-metastatic cells (red), metastatic cells (blue) and other types of cells (green) relative to each other. **C** Tumor progression trajectory analysis. The arrows show the line of the progression trajectory of gastric cancer (right), and the necroptosis score and EMT score, and immunity score along the trajectory region (left). **D** MDK-NCL signaling profile. Left: midkine (MDK) is enriched in the tumor region. Middle panel: nucleolin (NCL) is enriched in the stromal region. Right: MDK-NCL signaling is enriched at the tumor-stroma interface.

To further assess the spatial organization of GC, we performed spatial transcriptomic (ST) analysis on cryosections derived from fresh tumor samples of four patients (see Methods Section). Initially, the spatial boundaries of tumor and stromal regions were defined based on H&E staining and pathological assessment of the frozen sections (Fig. 7B). Following ST sequencing, we processed the data using the UMAP algorithm for dimensionality reduction and clustering, which categorized all spatial spots into 19 distinct clusters (Fig. 7A). To resolve cellular heterogeneity and characterize cell-type distributions within the spatial context, we performed deconvolution analysis using Cell2location (v0.1.4), which maps cell type information from a single-cell transcriptome onto ST cryosections, allowing for the estimation of the abundance of each cell type within spatially resolved tissue regions in a probabilistic manner. The resulting cell-type compositions were visualized as pie charts overlaid on the tissue image. This approach successfully preserved spatial tissue architecture while quantifying the relative abundance of distinct cell populations in both tumor and stromal regions. The deconvolution results showed strong agreement with independent histopathological annotations from H&E staining, supporting the reliability of our spatial data (Fig. 7B).

By integrating ST data with H&E staining, we further annotated the tissue into metastatic and non-metastatic regions. To investigate spatiotemporal dynamics of TME during tumor metastasis, we reconstructed a progression trajectory from non-metastatic to metastatic zones using SPATA2 (v3.1.3). Along this trajectory, we visualized computed scores for EMT, necroptosis, and cellular immunity. The results indicated a marked increase in both EMT and necroptosis scores, concomitant with a gradual decrease in immune activity along the metastatic trajectory (Fig. 7C). Spatial interaction analysis further revealed a distinct pattern: MDK ligand was highly expressed within tumor regions, whereas its receptor NCL was predominantly enriched in adjacent stromal cells (Fig. 7D).

Collectively, these spatial findings support our hypothesis that necroptosis may activate the MDK–NCL signaling axis via DAMP release, which in turn could mediate the establishment of an immunosuppressive microenvironment conducive to tumor metastasis.

## Discussion

In this study, we delineated a comprehensive transcriptomic profile of various cell types in GC and MLNs by scRNA-seq, which characterized the heterogeneity of both tumor cells and the tumor microenvironment in PT and MLN. We performed pseudo-time analysis on tumor cells revealed from PT and MLN and constructed a transcription trajectory with two branches. One branch showed lymph node metastasis potential based on cell origin. The results imply that there may be two tumor cell subclones in PT, one with lymph node metastasis potential and the other located at the primary site which is consistent with the genetic stability and transcriptional diversity of primary and metastatic tumor cells [31]. Notably, the metastatic branch was significantly enriched in the necroptosis pathway, suggesting that this form of programmed cell death may facilitate lymph node metastasis in GC — a finding that provides context for the dichotomous roles of necroptosis in both promoting and inhibiting metastasis [32, 33]. Although DAMPs can potentially stimulate anti-tumor immunity by alerting the immune system, they may also foster chronic inflammation and tumor progression under specific conditions [34, 35]. In GC, we propose a model wherein DAMPs released via necroptosis could initiate the MDK–NCL pathway, thereby facilitating pre-metastatic niche formation. However, the precise relationship between DAMP release and MDK–NCL activation requires further investigation.

By integrating single-cell and spatial transcriptomic approaches, our study overcomes limitations of previous works reliant solely on scRNA-seq for exploring LN metastasis mechanisms in GC [36]. We generated a more comprehensive spatial transcriptomic atlas at single-cell resolution, enabling deeper insights into the metastatic process. Through ST technology, we localized tumor-derived MDK and its stromal receptor NCL, and observed their co-enrichment at the tumor–stroma interface, suggesting spatial coordination of this signaling pathway with immune suppression. Spatial mapping further revealed that immune activity progressively declined with enhanced MDK–NCL signaling at the invasive front, indicating that metastatic cells can sculpt an immune-“cold” microenvironment. This supports the concept that tumor metastasis is a spatially coordinated process involving cross-talk between tumor and stromal cells³¹. Previous studies have implicated the MDK–NCL pathway in immune evasion across multiple malignancies [24, 27, 37]. Consistent with this, we found that MDK–NCL activity negatively correlated with ImmuneScore and StromalScore—as validated in the TCGA database—implying its role in suppressing anti-tumor immunity. Spatial mapping further revealed that immune activity progressively declined with enhanced MDK–NCL signaling at the invasive front, indicating that metastatic cells can sculpt an immune-“cold” microenvironment. This supports the concept that tumor metastasis is a spatially coordinated process involving cross-talk between tumor and stromal cells [21].

Several limitations of this study should be acknowledged. First, the small sample size (*n* = 4) necessitates validation in larger cohorts. Furthermore, it remains unclear whether necroptosis directly activates the MDK-NCL signaling pathway through DAMPs. In particular, although a significant correlation between CHMP3 expression and lymph node metastasis was observed in both TCGA data and our paired immunofluorescence samples, it is critical to note that these data indicate an association rather than causality. Additionally, the expression of other necroptosis-related proteins may also contribute to metastasis, and future studies should include a broader panel of markers to comprehensively evaluate the role of necroptosis in GC metastasis. Therefore, future studies should include a broader panel of markers to comprehensively evaluate the role of necroptosis in GC metastasis and validate these interactions through cellular and animal experiments to establish causal relationships and elucidate the underlying mechanisms.

## Materials and methods

### Ethical statement

This study was conducted in accordance with the Declaration of Helsinki and was approved by the Ethics Committee of Taizhou People’s Hospital Affiliated with Nanjing Medical University (Approval No.: KY 2023-133-01). Written informed consent was obtained from all participants prior to their inclusion in the study.

### Human specimens

This study enrolled four patients with gastric cancer in the Department of General Surgery of Taizhou People’s Hospital affiliated with Nanjing Medical University. A total of six fresh surgical specimens (four primary tumors and two paired metastatic lymph nodes) were collected. The resected gastric cancer tissues were sectioned into three pieces along the long axis: one piece was used for pathological diagnosis, one piece was used for scRNA-seq, and the other piece was used for spatial transcriptomics analysis. We also collected two paired metastatic lymph nodes and cut them into two parts: one for pathological diagnosis and the other for scRNA-seq. The pathologist confirmed the metastasis of the lymph nodes by cytological testing during the surgery and by paraffin sectioning after the surgery. The detailed clinical information was shown in the Supplementary Table S1.

### Tissue specimen collection

Paired paraffin-embedded tissue samples (including adjacent normal tissues, primary gastric cancer, and metastatic lymph nodes) were collected from 20 gastric cancer patients at Taizhou People’s Hospital Affiliated to Nanjing Medical University for immunofluorescence staining. None of the gastric cancer patients had received radiotherapy, chemotherapy, or other anticancer treatments prior to surgery.

### Tissue dissociation and single-cell preparation

Fresh GC tissues were placed in a Petri dish on wet ice containing cold PBS (without Ca² and Mg²) and rinsed to remove blood and debris. Tissues were then minced into approximately 0.5 mm³ pieces using a surgical scalpel and washed again with PBS. The minced tissues were transferred to a conical tube and digested in a dissociation cocktail comprising 0.35% collagenase IV, 2 mg/mL papain, and 120 U/mL DNase I in PBS. Digestion was performed at 37 °C for 20 min in a water bath shaker at 100 rpm. The reaction was quenched by adding an equal volume of PBS supplemented with 10% fetal bovine serum (FBS). The digested tissue was gently triturated 5–10 times with a wide-bore pipette tip to dissociate the cells without causing mechanical damage. The resulting cell suspension was sequentially filtered through 70-μm and 30-μm cell strainers and centrifuged at 300 g for 5 min at 4 °C. The cell pellet was resuspended in 100 μL of PBS containing 0.04% BSA. Erythrocytes were lysed by adding 1 mL of 1X RBC Lysis Buffer (MACS, 130-094-183) and incubating on ice for 10 min. After lysis, cells were centrifuged at 300 g for 5 minutes at 4 °C. Dead cells were removed using the Dead Cell Removal MicroBeads kit (MACS, 130-090-101) according to the manufacturer’s instructions. Briefly, the cell pellet was resuspended in 100 μL of Binding Buffer and incubated with the microbeads for 15 min at room temperature. The mixture was then applied to an MS Column (MACS, 130-042-201) placed in a magnetic field. The flow-through, containing live cells, was collected and centrifuged. The live cell pellet was washed twice with PBS containing 0.04% BSA. Cell viability and concentration were assessed using trypan blue exclusion on a Countess II Automated Cell Counter (Thermo Fisher Scientific). Only cell suspensions with viability >85% and a concentration of 700-1200 cells/μL were used for downstream library preparation.

### Single-cell library preparation and sequencing

Single-cell suspensions were loaded onto a Chromium Chip B according to the manufacturer’s protocol for the Chromium Single-Cell 3’ Reagent Kit (v3, 10x Genomics), targeting a recovery of approximately 8000 cells per sample. cDNA amplification and library construction were performed following the standard protocol. The resulting libraries were quantified using a Qubit fluorometer (Thermo Fisher Scientific) and assessed for quality using an Agilent 2100 Bioanalyzer. Libraries were sequenced on an Illumina NovaSeq 6000 platform by LC-Bio Technology Co., Ltd. (Hangzhou, China) using a paired-end 150 bp (PE150) read strategy, with a minimum sequencing depth of 20,000 reads per cell.

### Bioinformatics analysis

#### Data preprocessing and quality control

Raw sequencing data (BCL files) were demultiplexed and converted to FASTQ format using bcl2fastq software (v.5.0.1). The quality of the raw data was assessed using FastQC. Read alignment, filtering, barcode counting, and UMI counting were performed against the GRCh38 human reference genome using Cell Ranger (v.7.2.0, 10x Genomics).

#### Single-cell data analysis

The filtered gene-barcode matrices generated by Cell Ranger were imported into the Seurat package (version 4.1.0) [37] in R (v.4.4.2) for downstream analysis. Low-quality cells were filtered out using the following criteria: cells with fewer than 500 expressed genes or more than 25% of reads mapping to mitochondrial genes were excluded. Data normalization was performed using the LogNormalize method. Variable features were identified using the VST method. Data scaling was performed regressing out the effects of mitochondrial gene percentage. Principal component analysis (PCA) was conducted on the scaled data of variable features. The first 20 principal components (PCs), selected based on an Elbow plot, were used for graph-based clustering and non-linear dimensionality reduction (UMAP). Cell clusters were annotated into major types (T cells, B cells, Monocytes, Epithelial cells, Fibroblasts, Endothelial cells, Neutrophils, Dendritic cells, Mast cells, Smooth muscle cells) using a combination of automated annotation (SingleR package v.2.0.0) [38] and manual inspection of canonical marker gene expression.

#### Differential expression and functional enrichment

Differential gene expression analysis between clusters or conditions was performed using the FindMarkers or FindAllMarkers function in Seurat (version 4.1.0) with the Wilcoxon rank sum test. Genes with an adjusted *p* < 0.05 (Bonferroni correction) and an absolute log2(fold change) > 0.5 were considered significantly differentially expressed. Gene Ontology (GO) and Kyoto Encyclopedia of Genes and Genomes (KEGG) pathway enrichment analyses were performed on the differentially expressed genes using the clusterProfiler package (v3.14.0). Gene Set Enrichment Analysis (GSEA) was conducted using the Hallmark gene sets (v.7.5.1) from MSigDB. Gene set variation analysis (GSVA) analysis was performed using the “GSVA” R package (v1.40.0) [39], and the data sources were the KEGG and Reactome databases of MSigDB () and fgsea (v1.14.0). Reactome database (http://www.gsea-msigdb.org). Single-sample GSEA (ssGSEA) was used to calculate enrichment scores for KEGG, GO-BP and Reactome pathways using the GSVA package. Visualization was achieved by ggplot2 (v3.3.2).

#### CNV inferring

Copy number variation (CNV) [40] was inferred using the infercnv package (v.1.0.4) with immune cells (B cells, T cells) as the reference to evaluate CNV for each cell across different chromosomal regions.

#### Cell trajectory analysis

Pseudotime analysis was performed usingMonocle2 (v2.9.0) Genes with average expression >1 and differential dispersion (*q* < 0.01) were selected for trajectory construction. The DDRTree method was used for dimensionality reduction, and normal epithelial cells were set as the trajectory root.

#### Cell communication network analysis

Cell-cell communication networks were analyzed using CellChat (v.1.1.3) [26]. We quantify the strength of intercellular communication between cell populations through the ligand-receptor interaction mass action law. The analysis included identification of over-expressed ligands/receptors, calculation of communication probabilities, and aggregation of communication networks. Interactions were filtered for those involving at least 10 cells per cell group.

#### TCGA data analysis

Data were downloaded from the TCGA database (https://portal.gdc.cancer.gov) [41] and organized, including RNAseq data from the STAR pipeline of the TCGA-STAD project with TPM-formatted data extracted, as well as clinical data. Prognostic data were obtained from a Cell article 35. After data preprocessing, which involved removing normal samples and those without clinical information, and performing log₂ (Value + 1) transformation on the RNAseq data, samples were divided into CHMP3 high-expression and low-expression groups using the surv_cutpoint function in the survminer package. The proportional hazards (PH) assumption was tested using the survival package. Survival analysis was conducted using the Log-rank test and Cox regression when the PH assumption was satisfied, and survival curves were visualized with the survminer and ggplot2 packages. Meanwhile, the estimate package (version 1.0.13) was used to calculate the immune score, stromal score, and ESTIMATES score. Finally, Spearman correlation test was performed to evaluate the correlation between these scores and genes in the MDK pathway.

### Spatial transcriptomics analysis

#### Sample preparation and sectioning

Fresh gastric cancer and metastatic lymph node tissues were collected from surgical resections. Tissues were washed 2–3 times with cold phosphate-buffered saline (PBS) and trimmed to appropriate dimensions for embedding. Excess moisture was carefully removed using filter paper to prevent ice crystal formation during freezing. Tissues were embedded in Optimal Cutting Temperature (OCT) compound (Sakura Tissue-Tek, 4532, USA) in specific orientations to preserve anatomical structures. Serial sections of 10 μm thickness were prepared using a cryostat microtome (Leica CM1950) and mounted on Visium Spatial Tissue Optimization Slides (10x Genomics).

#### Library preparation and sequencing

The above samples were coronal sectioned at a thickness of 10 µm using a freezing microtome (Leica, CM1950). Tissue sections were fixed in 3.7% formaldehyde for 10 min at room temperature, followed by dehydration in isopropyl alcohol and air drying. In order to obtain suitable areas on the Visium space slides, we performed rapid H&E staining on a series of sections. Tissue fixation, HE staining and bright field imaging were used to establish optimal conditions for infiltration. Tissue sections were placed on Visium Spatial Tissue Optimized Slides (10x Genomics) covering six capture areas, one as a positive control and one as a negative control. Immediately after tissue staining, imaging and prepermeabilization, the tissue was permeabilized and reverse transcribed in situ. Spatial barcoding and library preparation were performed using the Visium Spatial Gene Expression kit (10x Genomics) according to manufacturer’s protocols. Libraries were sequenced on an Illumina NextSeq platform with paired-end 150 bp reads. Finally, library sequencing was performed on the Illumina NextSeq platform using paired-end sequencing. H&E-stained sections of each sample were carefully examined by two experienced pathologists to confirm pathology, and then manually annotated by trained pathologists to identify tumor, mesenchymal regions.

### Spatial transcriptome data analysis

#### Data preprocessing

Raw sequencing data were processed using Space Ranger (v.1.0.0, 10x Genomics) [42] for alignment to the GRCh38 reference genome and unique molecular identifier (UMI) counting. Tissue spots were automatically identified based on brightfield images, and spots overlapping with tissue sections were retained for downstream analysis. The filtered UMI count matrix was imported into R (v.4.2.2) for subsequent analysis. We also manually excluded points not detected by Space Ranger that were not covered by tissue and further filtered the UMI count matrix.

#### Data normalization and clustering

Spatial transcriptomics data were analyzed using the Seurat (v.4.1.0) and SPATA2 (v.3.1.3) packages. Data normalization was performed using SCTransform. Highly variable genes (*n* = 3000) were identified for each sample and used for principal component analysis. The first 30 principal components, determined by elbow plot analysis, were used for UMAP dimensionality reduction and graph-based clustering.

#### Cell type deconvolution

Using the first 30 principal components as input, Uniform Manifold Approximation and Projection (UMAP) dimensionality reduction was performed to visualize points and identify clusters. To identify molecular features associated with spatial locations within the tissue, differential expression was performed using Seurat based on pre-labeled anatomical regions within the tissue. These anatomical regions can be determined by unsupervised clustering or a priori knowledge. BMS scores were calculated for each ST point using the AddModuleScore() function and default parameters in Seurat, where the top 25–100 scores were significantly different between EDCs and other epithelial cells in the four tumor samples. To de-convolve cell types in scRNA-seq, the number of cells of each cell type was first down-sampled to 100 in the corresponding scRNA-seq data, and then SPOTlight [43] de-convolution was applied and the thirteen cell types were mapped to the ST segment. The probability of each cell type was set to less than 0.2 as noise, and then the highest probability was selected from all cell types as the recognized cell type.

#### Reverse convolution

Deconvolution was performed using the Cell2location software (0.1.4) to map the cell type information from the single-cell transcriptome to the empty transcriptome and calculate the percentage of cellular composition corresponding to each point based on the deconvolution results.

#### Trajectory analysis SPATA2

By the judgment of HE cases, the region of the null tissue was divided into, tumor metastasis area and tumor non-metastasis area. A trajectory from non-metastatic to metastatic was delineated by SPATA2 (3.1.3) software, and the expression and co-expression of ligand receptors in the tissue, EMT and apoptosis scores were obtained from addModuleScore software, and the R package ggsc (V 1.5.0).

### Immunofluorescence

Immunofluorescence staining was performed on paired tissue samples from 20 gastric cancer patients, including adjacent normal tissues, primary gastric cancer, and metastatic lymph nodes. The paraffin sections were deparaffinized with xylene and hydrated with gradient ethanol, and then antigenically repaired by high-pressure thermal repair method (sodium citrate buffer, pH 6.0, 95 °C for 15–20 min), followed by closure of the non-specific sites with 10% BSA. Rabbit anti-human CHMP3 antibody (1:200) was used for overnight incubation at 4 °C, respectively, and HRP-labeled secondary antibody (37 °C for 20 min) was conjugated to label the target proteins by TSA fluorescence signal amplification system (520 nm emission wavelength dye, 5–10 min), and the nuclei of the cells were re-stained by DAPI (1 μg/mL). All sections were subjected to image acquisition by laser confocal microscopy (Zeiss LSM 880, 40× oil microscope), and five fields of view were randomly selected for each sample, and fluorescence intensity and co-localization (Pearson coefficient) were analyzed using ImageJ software. Negative control (PBS substituted primary antibody) and positive control (known high expression tissue) were tested simultaneously to ensure the reliability of the results.

## Supplementary information

Support Table 1

Supporting Information

Data S1

Support Table 2

## Data Availability

The datasets used and analysed during the current study are available from the corresponding author on reasonable request.
