# Supplementary material for: Single-cell and spatial dissection of necroptosis spatiotemporal evolution driving lymph node metastasis in gastric cancer
Source: Cell Death Discov. 2025 Nov 17;11:535. doi: 10.1038/s41420-025-02815-z (PMC12623802; doi:10.1038/s41420-025-02815-z)
Supplement: Supplementary file 1 — Support Table 1 [file 41420_2025_2815_MOESM1_ESM.doc]

**Tables S1： Clinical characteristics of patients in the present study.**

| Patient ID | Age (year) | Sex | Tumor size  (cm) | Lymph nodes | Cancer type | TNM | Tissue |
| --- | --- | --- | --- | --- | --- | --- | --- |
| Patient1 | 66 | male | 4*3*1.5 | 7/45 | STAD | T3N3MO | Tumor Lymph nodes |
| Patient2 | 64 | male | 7*5*2.3 | 5/23 | GCA | T3N2M0 | Tumor  Lymph nodes |
| Patient3 | 72 | male | 3*2.5*0.5 | 1/37 | STAD | T1bN1M0 | Tumor |
| Patient4 | 77 | Female | 2.5*2.0*1.8 | 1/14 | STAD | T3N1MO | Tumor |
