# Supplementary material for: Single-cell and spatial dissection of necroptosis spatiotemporal evolution driving lymph node metastasis in gastric cancer
Source: Cell Death Discov. 2025 Nov 17;11:535. doi: 10.1038/s41420-025-02815-z (PMC12623802; doi:10.1038/s41420-025-02815-z)
Supplement: Supplementary file 2 — Supporting Information [file 41420_2025_2815_MOESM2_ESM.docx]

**Supporting Information**

**Single-cell and spatial transcriptomics reveal necroptosis-driven MDK-NCL signaling in lymph node metastasis of gastric cancer**

Yuhua Hu^#,2^, Honghong Zhang^#1^, Feng Shen^#1^, Xiaojun Zhao^#1^, Zhuowen Long^#3^, Jiale Wen^1,2^, Junxing Huang^*1,3^, Yan Chen^*1^, Qing Guo^*1^

**This supporting information includes:**

A:Table 1-2 B:Data S1 C:Figures. S1 to S4 D:Legends for S1 to S4

**Table 1.**Demographics and clinicopathological characteristics of the study population

**Table 2.**Results of single - gene Logistic regression analysis.

**Data S1.**The gene list acquired from ImmPort is utilized for the purpose of immunological scoring in the context of spatial transcriptomics.


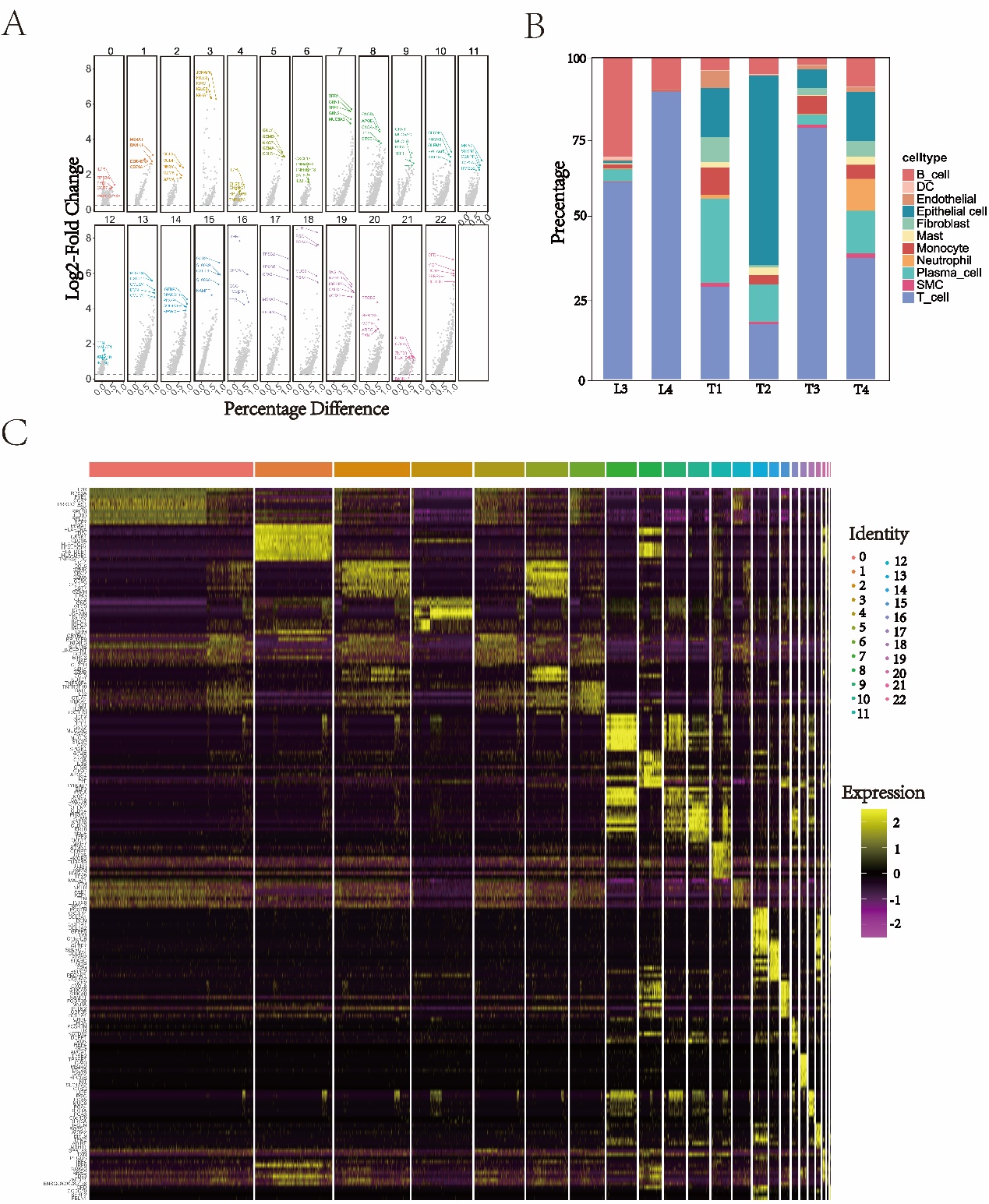


**Figure S1. Cell types identified by mark genes of sc-RNA seq clusters. (A)** The bar chart illustrates the relative proportions of various cell types in four primary tumors and their corresponding lymph nodes. Each cell type is represented by a distinct color. **(B)** Volcano plots shows the changes in expression levels of marker genes in different cell populations. The figure shows the log2 fold change and the difference in expression proportion for the top 5 marker genes in each sc-RNA seq cluster. **(C)** Expression levels of selected known marker genes across 50 599 unsorted cells illustrated in t-SNE plots from both primary cancer and lymph node tissue in gastric adenocarcinoma patients


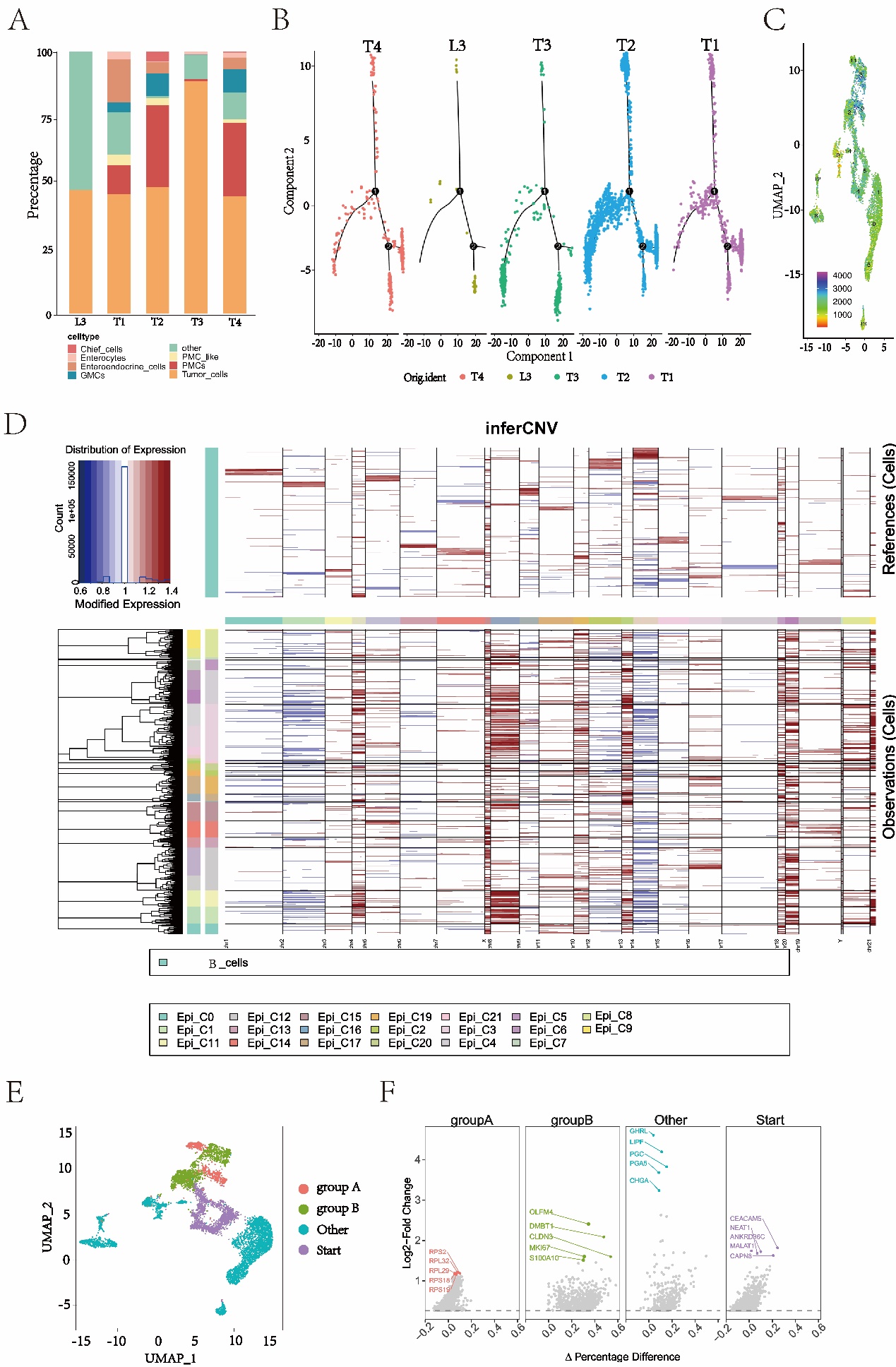


**Figure S2. Malignant epithelial cells identified by CNV levels.**

**(A)** The bar chart displays the reclassification of epithelial cell clusters in each lymph node and tumor sample. Each cell type is shown in a different color. **(B)** The scatter plot is utilized to illustrate the trajectories and distributions of cells originating from various tissues during the process of tumor metastasis. The points of different colors represent cells derived from different tissues. It is noteworthy that there are no tumor cells observed in the L4 MLN, a significant reason for which may be the small volume of the lymph tissue sample used for single-cell sequencing. Pathological data indicate that this lymph node had undergone metastasis. The lymph node was divided into two parts: one part was used for sequencing analysis, while the other was provided by the hospital for pathological reporting of normal patients. **(C)**The UMAP plot is used to visualize the results of CNV (copy number variation) analysis for different epithelial cell clusters, where the CNV levels of each cluster are distinguished by different colors. **(D)** The heat map shows the large-scale copy number variations (CNVs) of individual cells (rows) from several clusters, which are calculated based on the average expression of 100 genes surrounding each chromosome position (columns). Red: amplification; Blue: deletion. **(E)** The UMAP plot is used to illustrate the classification of 3403 gastric cancer cells from different branches of cellular trajectory analysis. Tumor cells from various branches are displayed in distinct colors. **(F)** The volcano plot reveals the changes in the expression levels of marker genes in different branches of gastric cancer cells. The expression level changes of the top 5 marker genes in each sc-RNA sequence cluster are depicted in the figure.


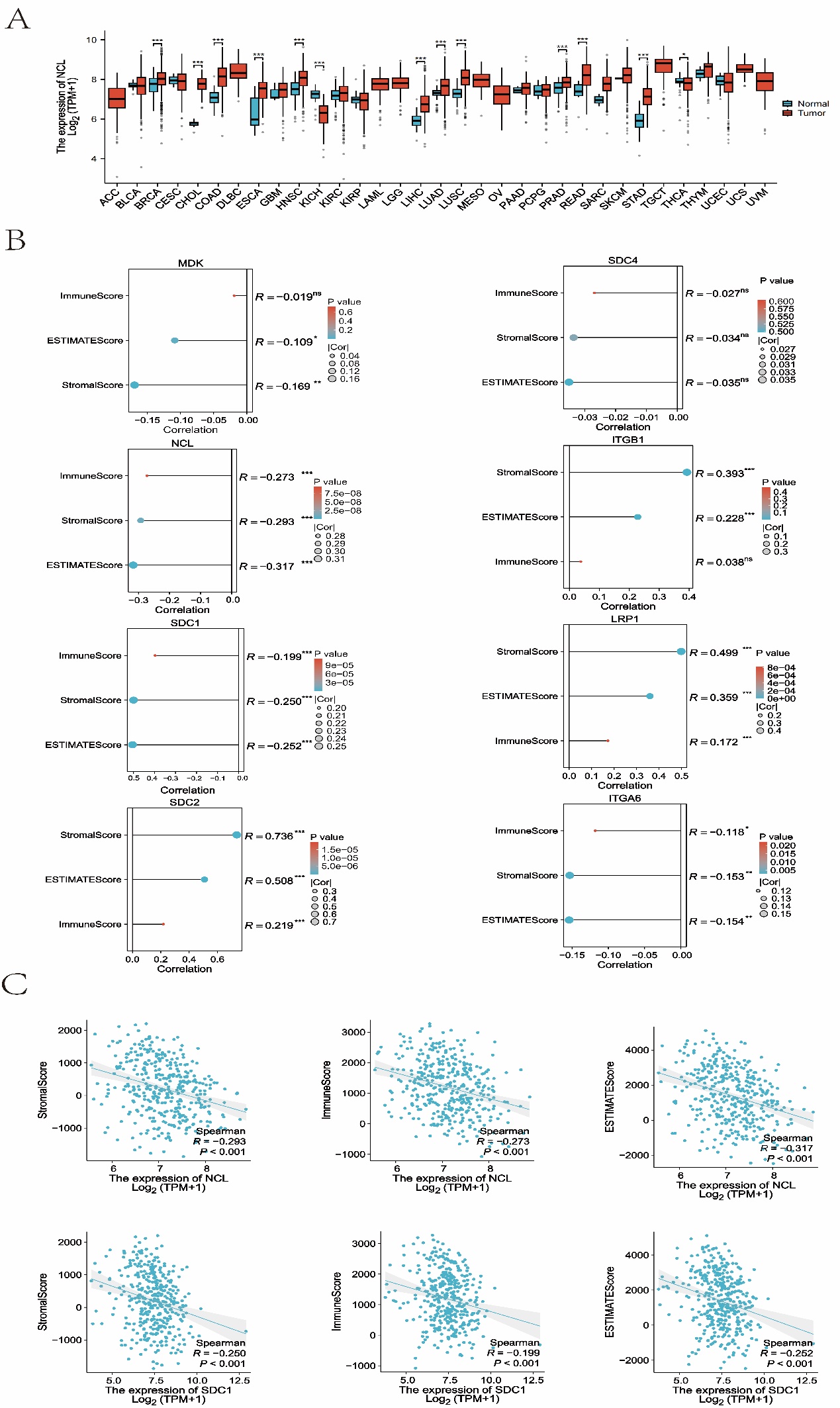


**Figure S3. The expression of the 8 genes involved in MK signaling network. (A)** The box plot illustrates the expression levels in pan-cancer tissues and normal endometrial tissues.**(B)**Lollipop chart showing the significant associations between the 8 genes and the scores estimated by “estimate” package with the TCGA data. ***P<0.001. **(C)** The scatter plot depicts the Spearman correlation between NCL and SDC1 with ImmuneScore or StromalScore


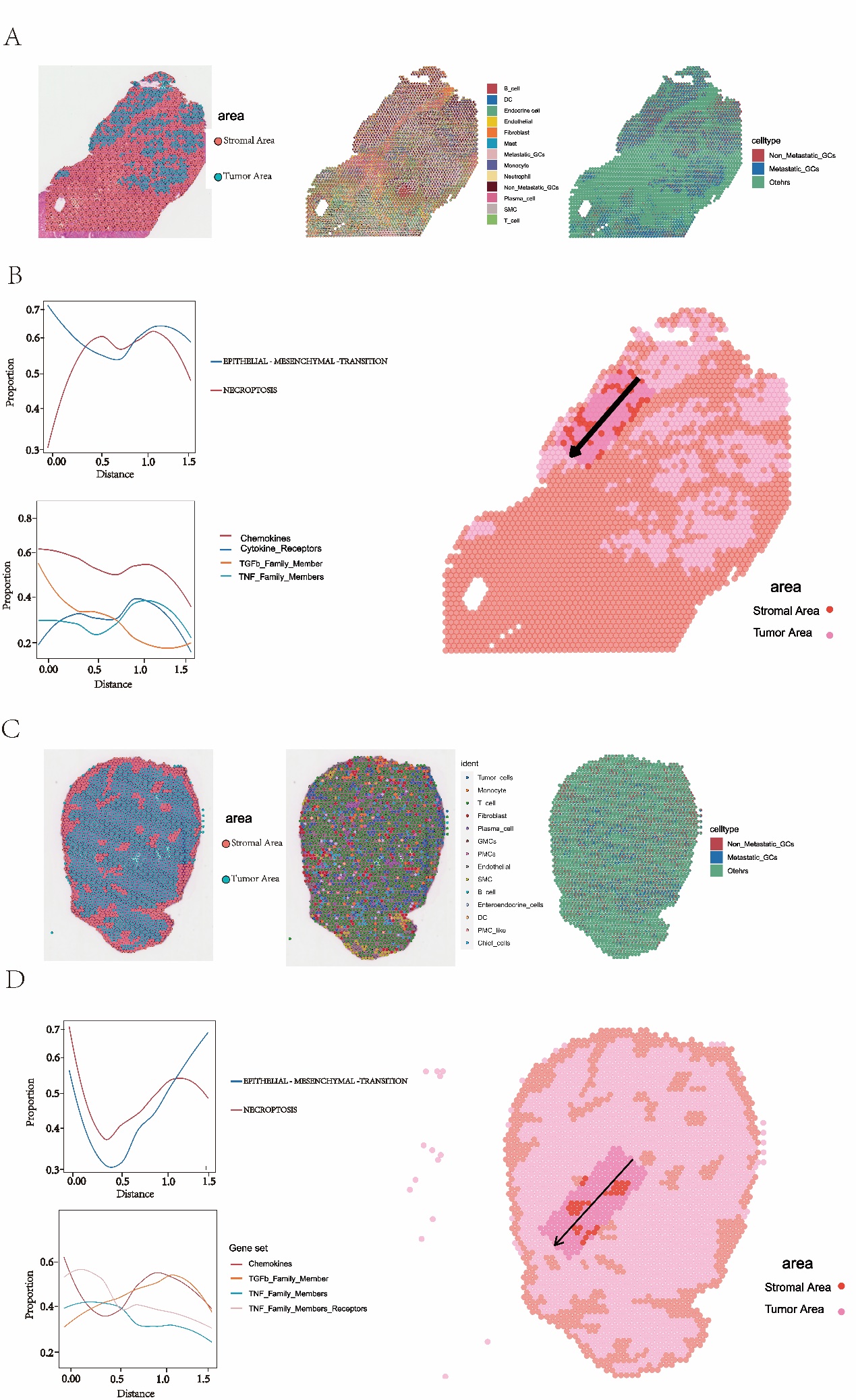


**Figure S4. Features of spatial transcriptomics dataset from primary tumors. (A)** H&E staining of T2 tissue section (left), with the deconvolution results of ST spots in four tumor samples showing the proportion of cell type composition (middle), and the spatial distribution patterns of tumor metastatic and non-metastatic regions (right). **(B)** The progression trajectory of gastric cancer (right), along with apoptosis scores, EMT scores, and immune scores based on the regions of cancer progression trajectory (left). **(C)** H&E staining of T3 tissue section (left), with the deconvolution results of ST spots in four tumor samples showing the proportion of cell type composition (middle), and the spatial distribution patterns of tumor metastatic and non-metastatic regions (right). **(D)** The progression trajectory of gastric cancer (right), along with apoptosis scores, EMT scores, and immune scores based on the regions of cancer progression trajectory (left).
