# Supplementary material for: Single-cell and spatial dissection of necroptosis spatiotemporal evolution driving lymph node metastasis in gastric cancer
Source: Cell Death Discov. 2025 Nov 17;11:535. doi: 10.1038/s41420-025-02815-z (PMC12623802; doi:10.1038/s41420-025-02815-z)
Supplement: Supplementary file 3 — Data S1 [file 41420_2025_2815_MOESM3_ESM.docx]

**Gene list obtained from ImmPort**

| **Symbol** | **Category** |
| --- | --- |
| AZGP1 | Antigen_Processing_and_Presentation |
| B2M | Antigen_Processing_and_Presentation |
| CALR | Antigen_Processing_and_Presentation |
| CANX | Antigen_Processing_and_Presentation |
| CD1A | Antigen_Processing_and_Presentation |
| CD1B | Antigen_Processing_and_Presentation |
| CD1C | Antigen_Processing_and_Presentation |
| CD1D | Antigen_Processing_and_Presentation |
| CD1E | Antigen_Processing_and_Presentation |
| CD4 | Antigen_Processing_and_Presentation |
| CD8A | Antigen_Processing_and_Presentation |
| CD8B | Antigen_Processing_and_Presentation |
| CD74 | Antigen_Processing_and_Presentation |
| CREB1 | Antigen_Processing_and_Presentation |
| CTSB | Antigen_Processing_and_Presentation |
| CTSE | Antigen_Processing_and_Presentation |
| CTSL1 | Antigen_Processing_and_Presentation |
| CTSS | Antigen_Processing_and_Presentation |
| FCER1G | Antigen_Processing_and_Presentation |
| FCGRT | Antigen_Processing_and_Presentation |
| PDIA3 | Antigen_Processing_and_Presentation |
| HFE | Antigen_Processing_and_Presentation |
| HLA-A | Antigen_Processing_and_Presentation |
| HLA-B | Antigen_Processing_and_Presentation |
| HLA-C | Antigen_Processing_and_Presentation |
| HLA-DMA | Antigen_Processing_and_Presentation |
| HLA-DMB | Antigen_Processing_and_Presentation |
| HLA-DOA | Antigen_Processing_and_Presentation |
| HLA-DOB | Antigen_Processing_and_Presentation |
| HLA-DPA1 | Antigen_Processing_and_Presentation |
| HLA-DPB1 | Antigen_Processing_and_Presentation |
| HLA-DQA1 | Antigen_Processing_and_Presentation |
| HLA-DQA2 | Antigen_Processing_and_Presentation |
| HLA-DQB1 | Antigen_Processing_and_Presentation |
| HLA-DRA | Antigen_Processing_and_Presentation |
| HLA-DRB1 | Antigen_Processing_and_Presentation |
| HLA-DRB3 | Antigen_Processing_and_Presentation |
| HLA-DRB4 | Antigen_Processing_and_Presentation |
| HLA-DRB5 | Antigen_Processing_and_Presentation |
| HLA-E | Antigen_Processing_and_Presentation |
| HLA-F | Antigen_Processing_and_Presentation |
| HLA-G | Antigen_Processing_and_Presentation |
| HLA-H | Antigen_Processing_and_Presentation |
| MR1 | Antigen_Processing_and_Presentation |
| HSPA1A | Antigen_Processing_and_Presentation |
| HSPA1B | Antigen_Processing_and_Presentation |
| HSPA1L | Antigen_Processing_and_Presentation |
| HSPA2 | Antigen_Processing_and_Presentation |
| HSPA4 | Antigen_Processing_and_Presentation |
| HSPA5 | Antigen_Processing_and_Presentation |

| HSPA6 | Antigen_Processing_and_Presentation |
| --- | --- |
| HSPA8 | Antigen_Processing_and_Presentation |
| HSP90AA1 | Antigen_Processing_and_Presentation |
| HSP90AB1 | Antigen_Processing_and_Presentation |
| ICAM1 | Antigen_Processing_and_Presentation |
| IFNA1 | Antigen_Processing_and_Presentation |
| IFNA2 | Antigen_Processing_and_Presentation |
| IFNA4 | Antigen_Processing_and_Presentation |
| IFNA5 | Antigen_Processing_and_Presentation |
| IFNA6 | Antigen_Processing_and_Presentation |
| IFNA7 | Antigen_Processing_and_Presentation |
| IFNA8 | Antigen_Processing_and_Presentation |
| IFNA10 | Antigen_Processing_and_Presentation |
| IFNA13 | Antigen_Processing_and_Presentation |
| IFNA14 | Antigen_Processing_and_Presentation |
| IFNA16 | Antigen_Processing_and_Presentation |
| IFNA17 | Antigen_Processing_and_Presentation |
| IFNA21 | Antigen_Processing_and_Presentation |
| IFNG | Antigen_Processing_and_Presentation |
| KIR2DL1 | Antigen_Processing_and_Presentation |
| KIR2DL2 | Antigen_Processing_and_Presentation |
| KIR2DL3 | Antigen_Processing_and_Presentation |
| KIR2DL4 | Antigen_Processing_and_Presentation |
| KIR2DS1 | Antigen_Processing_and_Presentation |
| KIR2DS3 | Antigen_Processing_and_Presentation |
| KIR2DS4 | Antigen_Processing_and_Presentation |
| KIR2DS5 | Antigen_Processing_and_Presentation |
| KIR3DL1 | Antigen_Processing_and_Presentation |
| KIR3DL2 | Antigen_Processing_and_Presentation |
| KLRC1 | Antigen_Processing_and_Presentation |
| KLRC2 | Antigen_Processing_and_Presentation |
| KLRC3 | Antigen_Processing_and_Presentation |
| KLRD1 | Antigen_Processing_and_Presentation |
| LTA | Antigen_Processing_and_Presentation |
| CIITA | Antigen_Processing_and_Presentation |
| MICA | Antigen_Processing_and_Presentation |
| MICB | Antigen_Processing_and_Presentation |
| NFYA | Antigen_Processing_and_Presentation |
| NFYB | Antigen_Processing_and_Presentation |
| NFYC | Antigen_Processing_and_Presentation |
| LGMN | Antigen_Processing_and_Presentation |
| PSMB8 | Antigen_Processing_and_Presentation |
| PSMC1 | Antigen_Processing_and_Presentation |
| PSMC2 | Antigen_Processing_and_Presentation |
| PSMC3 | Antigen_Processing_and_Presentation |
| PSMC4 | Antigen_Processing_and_Presentation |
| PSMC5 | Antigen_Processing_and_Presentation |
| PSMC6 | Antigen_Processing_and_Presentation |
| PSMD1 | Antigen_Processing_and_Presentation |
| PSMD2 | Antigen_Processing_and_Presentation |
| PSMD3 | Antigen_Processing_and_Presentation |
| PSMD4 | Antigen_Processing_and_Presentation |

| PSMD5 | Antigen_Processing_and_Presentation |
| --- | --- |
| PSMD7 | Antigen_Processing_and_Presentation |
| PSMD8 | Antigen_Processing_and_Presentation |
| PSMD10 | Antigen_Processing_and_Presentation |
| PSMD11 | Antigen_Processing_and_Presentation |
| PSMD13 | Antigen_Processing_and_Presentation |
| PSME1 | Antigen_Processing_and_Presentation |
| PSME1 | Antigen_Processing_and_Presentation |
| PSME2 | Antigen_Processing_and_Presentation |
| PSME2 | Antigen_Processing_and_Presentation |
| RELB | Antigen_Processing_and_Presentation |
| RFX5 | Antigen_Processing_and_Presentation |
| RFXAP | Antigen_Processing_and_Presentation |
| SLC10A2 | Antigen_Processing_and_Presentation |
| TAP1 | Antigen_Processing_and_Presentation |
| TAP2 | Antigen_Processing_and_Presentation |
| TAPBP | Antigen_Processing_and_Presentation |
| THBS1 | Antigen_Processing_and_Presentation |
| SHFM1 | Antigen_Processing_and_Presentation |
| KLRC4 | Antigen_Processing_and_Presentation |
| AP3B1 | Antigen_Processing_and_Presentation |
| RFXANK | Antigen_Processing_and_Presentation |
| PSMD6 | Antigen_Processing_and_Presentation |
| PSME3 | Antigen_Processing_and_Presentation |
| PSMD14 | Antigen_Processing_and_Presentation |
| CLEC4M | Antigen_Processing_and_Presentation |
| IFI30 | Antigen_Processing_and_Presentation |
| PROCR | Antigen_Processing_and_Presentation |
| ADRM1 | Antigen_Processing_and_Presentation |
| KIAA0368 | Antigen_Processing_and_Presentation |
| TRPC4AP | Antigen_Processing_and_Presentation |
| CD209 | Antigen_Processing_and_Presentation |
| UBXN1 | Antigen_Processing_and_Presentation |
| ERAP1 | Antigen_Processing_and_Presentation |
| TAPBPL | Antigen_Processing_and_Presentation |
| KIR2DL5A | Antigen_Processing_and_Presentation |
| ERAP2 | Antigen_Processing_and_Presentation |
| ULBP3 | Antigen_Processing_and_Presentation |
| ULBP2 | Antigen_Processing_and_Presentation |
| ULBP1 | Antigen_Processing_and_Presentation |
| KIR3DL3 | Antigen_Processing_and_Presentation |
| RAET1E | Antigen_Processing_and_Presentation |
| RAET1L | Antigen_Processing_and_Presentation |
| UBR1 | Antigen_Processing_and_Presentation |
| RAET1G | Antigen_Processing_and_Presentation |
| PDIA2 | Antigen_Processing_and_Presentation |
| HAMP | Antimicrobials |
| PI3 | Antimicrobials |
| CAMP | Antimicrobials |
| DEFB4 | Antimicrobials |
| PPBP | Antimicrobials |
| REG3G | Antimicrobials |

| CXCL14 | Antimicrobials |
| --- | --- |
| CXCL16 | Antimicrobials |
| SLPI | Antimicrobials |
| IL8 | Antimicrobials |
| CXCL10 | Antimicrobials |
| CXCL9 | Antimicrobials |
| CXCL5 | Antimicrobials |
| CXCL11 | Antimicrobials |
| CXCL6 | Antimicrobials |
| CXCL1 | Antimicrobials |
| CXCL12 | Antimicrobials |
| CXCL13 | Antimicrobials |
| CXCL2 | Antimicrobials |
| PF4 | Antimicrobials |
| XCL1 | Antimicrobials |
| CXCL3 | Antimicrobials |
| DEFB103A | Antimicrobials |
| CCL13 | Antimicrobials |
| CCL1 | Antimicrobials |
| DEFB1 | Antimicrobials |
| CCL8 | Antimicrobials |
| ELANE | Antimicrobials |
| DEFB103B | Antimicrobials |
| DEFA3 | Antimicrobials |
| DEFA1 | Antimicrobials |
| TMSB10 | Antimicrobials |
| DEFA6 | Antimicrobials |
| DEFA5 | Antimicrobials |
| DEFA4 | Antimicrobials |
| LCN2 | Antimicrobials |
| LCN1 | Antimicrobials |
| COLEC10 | Antimicrobials |
| BPI | Antimicrobials |
| S100A9 | Antimicrobials |
| S100A8 | Antimicrobials |
| DCD | Antimicrobials |
| LCN6 | Antimicrobials |
| S100A12 | Antimicrobials |
| HTN3 | Antimicrobials |
| LCN8 | Antimicrobials |
| LOC728358 | Antimicrobials |
| CCR10 | Antimicrobials |
| CELA1 | Antimicrobials |
| DEFB106A | Antimicrobials |
| PENK | Antimicrobials |
| BPIL2 | Antimicrobials |
| MMP12 | Antimicrobials |
| BPIL3 | Antimicrobials |
| LEAP2 | Antimicrobials |
| SFTPD | Antimicrobials |
| LCN9 | Antimicrobials |
| BPIL1 | Antimicrobials |

| PTGDS | Antimicrobials |
| --- | --- |
| TMSB4X | Antimicrobials |
| PGLYRP1 | Antimicrobials |
| ZC3HAV1 | Antimicrobials |
| TMSB15A | Antimicrobials |
| S100B | Antimicrobials |
| S100A13 | Antimicrobials |
| S100A6 | Antimicrobials |
| DEFB119 | Antimicrobials |
| DEFB107A | Antimicrobials |
| DEFB105A | Antimicrobials |
| SERPIND1 | Antimicrobials |
| DEFB129 | Antimicrobials |
| DEFB127 | Antimicrobials |
| S100P | Antimicrobials |
| S100A7 | Antimicrobials |
| DEFB104A | Antimicrobials |
| DEFB126 | Antimicrobials |
| DEFB106B | Antimicrobials |
| DEFB104B | Antimicrobials |
| DEFB107B | Antimicrobials |
| PGLYRP3 | Antimicrobials |
| PGLYRP2 | Antimicrobials |
| S100A10 | Antimicrobials |
| S100A2 | Antimicrobials |
| DEFB125 | Antimicrobials |
| DEFB123 | Antimicrobials |
| DEFB105B | Antimicrobials |
| DEFB132 | Antimicrobials |
| C20orf185 | Antimicrobials |
| LCN12 | Antimicrobials |
| PGLYRP4 | Antimicrobials |
| S100A11 | Antimicrobials |
| S100A5 | Antimicrobials |
| S100A3 | Antimicrobials |
| S100A1 | Antimicrobials |
| DEFB128 | Antimicrobials |
| DEFB108B | Antimicrobials |
| HTN1 | Antimicrobials |
| LMBR1L | Antimicrobials |
| S100A7A | Antimicrobials |
| DEFB118 | Antimicrobials |
| COLEC12 | Antimicrobials |
| TMSB4Y | Antimicrobials |
| DEFB131 | Antimicrobials |
| DEFB134 | Antimicrobials |
| DEFB130 | Antimicrobials |
| DEFB124 | Antimicrobials |
| DEFB121 | Antimicrobials |
| DEFB116 | Antimicrobials |
| DEFB115 | Antimicrobials |
| DEFB114 | Antimicrobials |

| DEFB113 | Antimicrobials |
| --- | --- |
| DEFB112 | Antimicrobials |
| DEFB110 | Antimicrobials |
| TMSB15B | Antimicrobials |
| DEFB133 | Antimicrobials |
| S100Z | Antimicrobials |
| MAVS | Antimicrobials |
| TMSL3 | Antimicrobials |
| S100A14 | Antimicrobials |
| LCN10 | Antimicrobials |
| S100A16 | Antimicrobials |
| DEFB137 | Antimicrobials |
| DEFB136 | Antimicrobials |
| DEFB117 | Antimicrobials |
| DEFB111 | Antimicrobials |
| ZC3HAV1L | Antimicrobials |
| S100A7L2 | Antimicrobials |
| LOC731414 | Antimicrobials |
| LOC730963 | Antimicrobials |
| COLEC2 | Antimicrobials |
| DEFB4P | Antimicrobials |
| C20orf186 | Antimicrobials |
| IFNAR1 | Antimicrobials |
| AZU1 | Antimicrobials |
| LOC729523 | Antimicrobials |
| LOC100130154 | Antimicrobials |
| LOC100134379 | Antimicrobials |
| LOC100134289 | Antimicrobials |
| LOC100129216 | Antimicrobials |
| DEFA1A3 | Antimicrobials |
| LOC100131433 | Antimicrobials |
| LCN1L1 | Antimicrobials |
| S100G | Antimicrobials |
| LOC648637 | Antimicrobials |
| LOC100130969 | Antimicrobials |
| LOC100133267 | Antimicrobials |
| LOC100133128 | Antimicrobials |
| LOC100128174 | Antimicrobials |
| TCHHL1 | Antimicrobials |
| TINAGL1 | Antimicrobials |
| IFNGR1 | Antimicrobials |
| SLC22A17 | Antimicrobials |
| WFIKKN1 | Antimicrobials |
| WFDC2 | Antimicrobials |
| IL6 | Antimicrobials |
| UMODL1 | Antimicrobials |
| TGFB1 | Antimicrobials |
| PF4V1 | Antimicrobials |
| MMP9 | Antimicrobials |
| KAL1 | Antimicrobials |
| TLR4 | Antimicrobials |
| IFNG | Antimicrobials |

| SPAG11B | Antimicrobials |
| --- | --- |
| A2M | Antimicrobials |
| CTSL1 | Antimicrobials |
| NFKB1 | Antimicrobials |
| APOBEC3G | Antimicrobials |
| FABP6 | Antimicrobials |
| NOD2 | Antimicrobials |
| MBL2 | Antimicrobials |
| SFTPA1B | Antimicrobials |
| RBP1 | Antimicrobials |
| TLR2 | Antimicrobials |
| SLC40A1 | Antimicrobials |
| PLAU | Antimicrobials |
| IL1B | Antimicrobials |
| PAEP | Antimicrobials |
| HFE2 | Antimicrobials |
| MUC5AC | Antimicrobials |
| CTSS | Antimicrobials |
| OBP2A | Antimicrobials |
| PLTP | Antimicrobials |
| MX1 | Antimicrobials |
| DDX58 | Antimicrobials |
| IL29 | Antimicrobials |
| IRF3 | Antimicrobials |
| SFTPA2 | Antimicrobials |
| SFTPA2B | Antimicrobials |
| LPA | Antimicrobials |
| LBP | Antimicrobials |
| RBP4 | Antimicrobials |
| SFTPA1 | Antimicrobials |
| NOX4 | Antimicrobials |
| LTF | Antimicrobials |
| IFNB1 | Antimicrobials |
| RBP5 | Antimicrobials |
| FABP7 | Antimicrobials |
| FABP5 | Antimicrobials |
| FABP3 | Antimicrobials |
| FABP2 | Antimicrobials |
| FABP4 | Antimicrobials |
| R3HDML | Antimicrobials |
| C20orf71 | Antimicrobials |
| C20orf114 | Antimicrobials |
| OASL | Antimicrobials |
| CRABP2 | Antimicrobials |
| CRABP1 | Antimicrobials |
| RBP7 | Antimicrobials |
| DUOX1 | Antimicrobials |
| OBP2B | Antimicrobials |
| RBP2 | Antimicrobials |
| LCN15 | Antimicrobials |
| CETP | Antimicrobials |
| FABP12 | Antimicrobials |

| FABP9 | Antimicrobials |
| --- | --- |
| PLUNC | Antimicrobials |
| LCNL1 | Antimicrobials |
| C8G | Antimicrobials |
| SPAG11A | Antimicrobials |
| PI15 | Antimicrobials |
| NOX1 | Antimicrobials |
| PMP2 | Antimicrobials |
| APOD | Antimicrobials |
| ORM2 | Antimicrobials |
| ORM1 | Antimicrobials |
| TNF | Antimicrobials |
| CTSG | Antimicrobials |
| PRTN3 | Antimicrobials |
| MAPK1 | Antimicrobials |
| PML | Antimicrobials |
| AEN | Antimicrobials |
| CYBB | Antimicrobials |
| C20orf70 | Antimicrobials |
| ISG20 | Antimicrobials |
| BCL3 | Antimicrobials |
| ISG20L2 | Antimicrobials |
| NOX5 | Antimicrobials |
| NOX3 | Antimicrobials |
| DUOX2 | Antimicrobials |
| TLR3 | Antimicrobials |
| TFRC | Antimicrobials |
| IFIH1 | Antimicrobials |
| LRP1 | Antimicrobials |
| TRIM5 | Antimicrobials |
| IDO1 | Antimicrobials |
| GDF15 | Antimicrobials |
| NEDD4 | Antimicrobials |
| ADIPOQ | Antimicrobials |
| STAT3 | Antimicrobials |
| STAT1 | Antimicrobials |
| IL28A | Antimicrobials |
| SOCS3 | Antimicrobials |
| SEMG1 | Antimicrobials |
| TNFSF10 | Antimicrobials |
| CCL20 | Antimicrobials |
| SOCS1 | Antimicrobials |
| RNASEL | Antimicrobials |
| IRF1 | Antimicrobials |
| IL15 | Antimicrobials |
| APOBEC3F | Antimicrobials |
| RARRES3 | Antimicrobials |
| CHIT1 | Antimicrobials |
| IFNA1 | Antimicrobials |
| CD40 | Antimicrobials |
| TLR7 | Antimicrobials |
| PPIA | Antimicrobials |

| HFE | Antimicrobials |
| --- | --- |
| ZYX | Antimicrobials |
| NLRX1 | Antimicrobials |
| PGC | Antimicrobials |
| VEGFA | Antimicrobials |
| IKBKE | Antimicrobials |
| ISG15 | Antimicrobials |
| DHX58 | Antimicrobials |
| TNFAIP3 | Antimicrobials |
| TFR2 | Antimicrobials |
| FCN2 | Antimicrobials |
| MUC4 | Antimicrobials |
| F2R | Antimicrobials |
| ELN | Antimicrobials |
| IL27 | Antimicrobials |
| MAPT | Antimicrobials |
| LYZ | Antimicrobials |
| CCL5 | Antimicrobials |
| LEP | Antimicrobials |
| CYLD | Antimicrobials |
| KLKB1 | Antimicrobials |
| CST4 | Antimicrobials |
| CSRP1 | Antimicrobials |
| MAPK14 | Antimicrobials |
| JUN | Antimicrobials |
| ITGAV | Antimicrobials |
| IRF5 | Antimicrobials |
| CCR6 | Antimicrobials |
| IL12B | Antimicrobials |
| TLR8 | Antimicrobials |
| GNLY | Antimicrobials |
| CD81 | Antimicrobials |
| EIF2AK2 | Antimicrobials |
| APOM | Antimicrobials |
| CACYBP | Antimicrobials |
| NOD1 | Antimicrobials |
| MAPK8 | Antimicrobials |
| MAPK3 | Antimicrobials |
| BST2 | Antimicrobials |
| BPHL | Antimicrobials |
| PLA2G2A | Antimicrobials |
| GRN | Antimicrobials |
| NEWENTRY | Antimicrobials |
| PDGFRA | Antimicrobials |
| GNAI1 | Antimicrobials |
| WNT5A | Antimicrobials |
| FURIN | Antimicrobials |
| ADAR | Antimicrobials |
| TYK2 | Antimicrobials |
| NOS2 | Antimicrobials |
| TRAF3 | Antimicrobials |
| TPT1 | Antimicrobials |

| TPM2 | Antimicrobials |
| --- | --- |
| NEO1 | Antimicrobials |
| AHNAK | Antimicrobials |
| TLR1 | Antimicrobials |
| TK2 | Antimicrobials |
| PRDX2 | Antimicrobials |
| MX2 | Antimicrobials |
| FGF2 | Antimicrobials |
| FGA | Antimicrobials |
| TCF7L2 | Antimicrobials |
| F2RL1 | Antimicrobials |
| DAK | Antimicrobials |
| MSR1 | Antimicrobials |
| NFKBIZ | Antimicrobials |
| LMBR1 | Antimicrobials |
| SPINLW1 | Antimicrobials |
| SRC | Antimicrobials |
| MPO | Antimicrobials |
| ELAVL1 | Antimicrobials |
| ROBO3 | Antimicrobials |
| SP1 | Antimicrobials |
| SOD1 | Antimicrobials |
| PDF | Antimicrobials |
| DLL4 | Antimicrobials |
| ECD | Antimicrobials |
| SLC11A1 | Antimicrobials |
| DMBT1 | Antimicrobials |
| TMEM173 | Antimicrobials |
| SKIV2L | Antimicrobials |
| SEMG2 | Antimicrobials |
| LTA | Antimicrobials |
| DES | Antimicrobials |
| DCK | Antimicrobials |
| DAXX | Antimicrobials |
| TNFRSF10A | Antimicrobials |
| TNFRSF10B | Antimicrobials |
| EED | Antimicrobials |
| CCL4 | Antimicrobials |
| LIMS1 | Antimicrobials |
| LALBA | Antimicrobials |
| APOBEC3H | Antimicrobials |
| TMPRSS6 | Antimicrobials |
| SPINK5 | Antimicrobials |
| MARCO | Antimicrobials |
| BECN1 | Antimicrobials |
| TNFSF11 | Antimicrobials |
| KNG1 | Antimicrobials |
| CSK | Antimicrobials |
| KLRK1 | Antimicrobials |
| KCNH2 | Antimicrobials |
| JUND | Antimicrobials |
| JAK1 | Antimicrobials |

| CREB1 | Antimicrobials |
| --- | --- |
| CLDN4 | Antimicrobials |
| CCL28 | Antimicrobials |
| RNASE3 | Antimicrobials |
| RN7SL1 | Antimicrobials |
| IRF7 | Antimicrobials |
| IREB2 | Antimicrobials |
| ILK | Antimicrobials |
| IL18 | Antimicrobials |
| IL17A | Antimicrobials |
| LTB4R | Antimicrobials |
| APOBEC3A | Antimicrobials |
| MASP2 | Antimicrobials |
| TRIM27 | Antimicrobials |
| RELA | Antimicrobials |
| IL7R | Antimicrobials |
| IL1A | Antimicrobials |
| PTX3 | Antimicrobials |
| IFNAR2 | Antimicrobials |
| IFN1@ | Antimicrobials |
| SYTL1 | Antimicrobials |
| APOBEC3C | Antimicrobials |
| DDX17 | Antimicrobials |
| PTGS2 | Antimicrobials |
| HTR1A | Antimicrobials |
| SEPT-7 | Antimicrobials |
| CD40LG | Antimicrobials |
| CD14 | Antimicrobials |
| CD8A | Antimicrobials |
| CD4 | Antimicrobials |
| MASP1 | Antimicrobials |
| PROC | Antimicrobials |
| MAP2K2 | Antimicrobials |
| MAP2K1 | Antimicrobials |
| HRG | Antimicrobials |
| NDRG1 | Antimicrobials |
| IRF9 | Antimicrobials |
| TRIM22 | Antimicrobials |
| LANCL1 | Antimicrobials |
| PPP4C | Antimicrobials |
| HMOX1 | Antimicrobials |
| HMGB1 | Antimicrobials |
| HLA-B | Antimicrobials |
| RNASE7 | Antimicrobials |
| ABCC4 | Antimicrobials |
| HGF | Antimicrobials |
| HDAC1 | Antimicrobials |
| IL28RA | Antimicrobials |
| PLSCR1 | Antimicrobials |
| B2M | Antimicrobials |
| BACH2 | Antimicrobials |
| TANK | Antimicrobials |

| PIK3CG | Antimicrobials |
| --- | --- |
| ARRB1 | Antimicrobials |
| RSAD2 | Antimicrobials |
| STAB2 | Antimicrobials |
| TBK1 | Antimicrobials |
| PDYN | Antimicrobials |
| PDGFRB | Antimicrobials |
| PDCD1 | Antimicrobials |
| PCSK2 | Antimicrobials |
| PCSK1 | Antimicrobials |
| ARG2 | Antimicrobials |
| AQP9 | Antimicrobials |
| FASLG | Antimicrobials |
| APOH | Antimicrobials |
| BIRC5 | Antimicrobials |
| ANXA6 | Antimicrobials |
| IL22 | Antimicrobials |
| VTN | Antimicrobials |
| VIM | Antimicrobials |
| VCAM1 | Antimicrobials |
| PRDX1 | Antimicrobials |
| GFAP | Antimicrobials |
| GBP2 | Antimicrobials |
| ALB | Antimicrobials |
| SLC29A3 | Antimicrobials |
| OAS1 | Antimicrobials |
| AGER | Antimicrobials |
| UNC93B1 | Antimicrobials |
| TNFSF4 | Antimicrobials |
| NOS1 | Antimicrobials |
| ACTG1 | Antimicrobials |
| ACTA1 | Antimicrobials |
| ACO1 | Antimicrobials |
| SERPINA3 | Antimicrobials |
| IL8RA | Antimicrobials |
| CCL15 | Antimicrobials |
| CCL14 | Antimicrobials |
| CCL4 | Antimicrobials |
| CCL16 | Antimicrobials |
| CCL19 | Antimicrobials |
| CCL13 | Antimicrobials |
| CCL18 | Antimicrobials |
| CCL17 | Antimicrobials |
| CCL26 | Antimicrobials |
| CCL22 | Antimicrobials |
| CCR3 | Antimicrobials |
| CCL28 | Antimicrobials |
| CCL4L2 | Antimicrobials |
| CCBP2 | Antimicrobials |
| CCR7 | Antimicrobials |
| CCL27 | Antimicrobials |
| CCR8 | Antimicrobials |

| CCRL1 | Antimicrobials |
| --- | --- |
| CCR10 | Antimicrobials |
| CCL2 | Antimicrobials |
| CCL21 | Antimicrobials |
| CCL7 | Antimicrobials |
| CCL5 | Antimicrobials |
| CCL3 | Antimicrobials |
| CCL20 | Antimicrobials |
| CCL11 | Antimicrobials |
| CCR5 | Antimicrobials |
| CCL23 | Antimicrobials |
| CCL25 | Antimicrobials |
| CCL1 | Antimicrobials |
| CCL3L3 | Antimicrobials |
| CCL4L1 | Antimicrobials |
| CXCL12 | Antimicrobials |
| XCL1 | Antimicrobials |
| CCL8 | Antimicrobials |
| CCL3L1 | Antimicrobials |
| CCR1 | Antimicrobials |
| CCL24 | Antimicrobials |
| XCL2 | Antimicrobials |
| CXCL1 | Antimicrobials |
| CXCL10 | Antimicrobials |
| CXCR4 | Antimicrobials |
| CXCL2 | Antimicrobials |
| CXCR6 | Antimicrobials |
| CCR4 | Antimicrobials |
| CXCL11 | Antimicrobials |
| FAM19A5 | Antimicrobials |
| FAM19A3 | Antimicrobials |
| FAM19A4 | Antimicrobials |
| FAM19A1 | Antimicrobials |
| FAM19A2 | Antimicrobials |
| CCL14-CCL15 | Antimicrobials |
| IL6 | Antimicrobials |
| TNF | Antimicrobials |
| IL1B | Antimicrobials |
| IL18 | Antimicrobials |
| PTK2B | Antimicrobials |
| VEGFA | Antimicrobials |
| IL4 | Antimicrobials |
| CDH1 | Antimicrobials |
| CD40 | Antimicrobials |
| DEFB103A | Antimicrobials |
| F2RL1 | Antimicrobials |
| MMP9 | Antimicrobials |
| LTBP1 | Antimicrobials |
| DEFB4 | Antimicrobials |
| TNFSF10 | Antimicrobials |
| IL13 | Antimicrobials |
| IL10 | Antimicrobials |

| IL2 | Antimicrobials |
| --- | --- |
| PPARG | Antimicrobials |
| FGR | Antimicrobials |
| MIF | Antimicrobials |
| CRP | Antimicrobials |
| JAK2 | Antimicrobials |
| IL1A | Antimicrobials |
| PTK2 | Antimicrobials |
| PTGDR | Antimicrobials |
| CD86 | Antimicrobials |
| HCK | Antimicrobials |
| ARRB1 | Antimicrobials |
| GNAI1 | Antimicrobials |
| VDR | Antimicrobials |
| OLR1 | Antimicrobials |
| ADRBK1 | Antimicrobials |
| TXK | Antimicrobials |
| RNASE2 | Antimicrobials |
| CD79A | BCRSignalingPathway |
| CD79B | BCRSignalingPathway |
| LYN | BCRSignalingPathway |
| SYK | BCRSignalingPathway |
| BTK | BCRSignalingPathway |
| BLNK | BCRSignalingPathway |
| VAV3 | BCRSignalingPathway |
| VAV1 | BCRSignalingPathway |
| VAV2 | BCRSignalingPathway |
| RAC1 | BCRSignalingPathway |
| RAC2 | BCRSignalingPathway |
| RAC3 | BCRSignalingPathway |
| PPP3CA | BCRSignalingPathway |
| PPP3CB | BCRSignalingPathway |
| PPP3CC | BCRSignalingPathway |
| CHP | BCRSignalingPathway |
| PPP3R1 | BCRSignalingPathway |
| PPP3R2 | BCRSignalingPathway |
| CHP2 | BCRSignalingPathway |
| NFAT5 | BCRSignalingPathway |
| NFATC1 | BCRSignalingPathway |
| NFATC2 | BCRSignalingPathway |
| NFATC3 | BCRSignalingPathway |
| NFATC4 | BCRSignalingPathway |
| HRAS | BCRSignalingPathway |
| KRAS | BCRSignalingPathway |
| NRAS | BCRSignalingPathway |
| FOS | BCRSignalingPathway |
| JUN | BCRSignalingPathway |
| CARD11 | BCRSignalingPathway |
| BCL10 | BCRSignalingPathway |
| MALT1 | BCRSignalingPathway |
| CHUK | BCRSignalingPathway |
| IKBKB | BCRSignalingPathway |

| IKBKG | BCRSignalingPathway |
| --- | --- |
| NFKB1 | BCRSignalingPathway |
| RELA | BCRSignalingPathway |
| NFKBIA | BCRSignalingPathway |
| NFKBIB | BCRSignalingPathway |
| NFKBIE | BCRSignalingPathway |
| CD81 | BCRSignalingPathway |
| CD19 | BCRSignalingPathway |
| CR2 | BCRSignalingPathway |
| PIK3R5 | BCRSignalingPathway |
| PIK3R1 | BCRSignalingPathway |
| PIK3R2 | BCRSignalingPathway |
| PIK3R3 | BCRSignalingPathway |
| PIK3CA | BCRSignalingPathway |
| PIK3CB | BCRSignalingPathway |
| PIK3CD | BCRSignalingPathway |
| PIK3CG | BCRSignalingPathway |
| AKT3 | BCRSignalingPathway |
| AKT1 | BCRSignalingPathway |
| AKT2 | BCRSignalingPathway |
| GSK3B | BCRSignalingPathway |
| INPP5D | BCRSignalingPathway |
| CD22 | BCRSignalingPathway |
| CD72 | BCRSignalingPathway |
| PTPN6 | BCRSignalingPathway |
| LILRB3 | BCRSignalingPathway |
| FCGR2B | BCRSignalingPathway |
| RASGRP3 | BCRSignalingPathway |
| PLCG2 | BCRSignalingPathway |
| PRKCB | BCRSignalingPathway |
| IFITM1 | BCRSignalingPathway |
| IGH@ | BCRSignalingPathway |
| IGHA1 | BCRSignalingPathway |
| IGHA2 | BCRSignalingPathway |
| IGHD | BCRSignalingPathway |
| IGHD@ | BCRSignalingPathway |
| IGHD1-1 | BCRSignalingPathway |
| IGHD1-14 | BCRSignalingPathway |
| IGHD1-20 | BCRSignalingPathway |
| IGHD1-26 | BCRSignalingPathway |
| IGHD1-7 | BCRSignalingPathway |
| IGHD2-15 | BCRSignalingPathway |
| IGHD2-2 | BCRSignalingPathway |
| IGHD2-21 | BCRSignalingPathway |
| IGHD2-8 | BCRSignalingPathway |
| IGHD3-10 | BCRSignalingPathway |
| IGHD3-16 | BCRSignalingPathway |
| IGHD3-22 | BCRSignalingPathway |
| IGHD3-3 | BCRSignalingPathway |
| IGHD3-9 | BCRSignalingPathway |
| IGHD4-11 | BCRSignalingPathway |
| IGHD4-17 | BCRSignalingPathway |

| IGHD4-23 | BCRSignalingPathway |
| --- | --- |
| IGHD4-4 | BCRSignalingPathway |
| IGHD5-12 | BCRSignalingPathway |
| IGHD5-18 | BCRSignalingPathway |
| IGHD5-24 | BCRSignalingPathway |
| IGHD5-5 | BCRSignalingPathway |
| IGHD6-13 | BCRSignalingPathway |
| IGHD6-19 | BCRSignalingPathway |
| IGHD6-25 | BCRSignalingPathway |
| IGHD6-6 | BCRSignalingPathway |
| IGHD7-27 | BCRSignalingPathway |
| IGHE | BCRSignalingPathway |
| IGHG1 | BCRSignalingPathway |
| IGHG2 | BCRSignalingPathway |
| IGHG3 | BCRSignalingPathway |
| IGHG4 | BCRSignalingPathway |
| IGHJ@ | BCRSignalingPathway |
| IGHJ1 | BCRSignalingPathway |
| IGHJ2 | BCRSignalingPathway |
| IGHJ3 | BCRSignalingPathway |
| IGHJ4 | BCRSignalingPathway |
| IGHJ5 | BCRSignalingPathway |
| IGHJ6 | BCRSignalingPathway |
| IGHM | BCRSignalingPathway |
| IGHV@ | BCRSignalingPathway |
| IGHV1-18 | BCRSignalingPathway |
| IGHV1-2 | BCRSignalingPathway |
| IGHV1-24 | BCRSignalingPathway |
| IGHV1-3 | BCRSignalingPathway |
| IGHV1-45 | BCRSignalingPathway |
| IGHV1-46 | BCRSignalingPathway |
| IGHV1-58 | BCRSignalingPathway |
| IGHV1-69 | BCRSignalingPathway |
| IGHV1-8 | BCRSignalingPathway |
| IGHV1-C | BCRSignalingPathway |
| IGHV1-F | BCRSignalingPathway |
| IGHV2-26 | BCRSignalingPathway |
| IGHV2-5 | BCRSignalingPathway |
| IGHV2-70 | BCRSignalingPathway |
| IGHV3-11 | BCRSignalingPathway |
| IGHV3-13 | BCRSignalingPathway |
| IGHV3-15 | BCRSignalingPathway |
| IGHV3-16 | BCRSignalingPathway |
| IGHV3-20 | BCRSignalingPathway |
| IGHV3-21 | BCRSignalingPathway |
| IGHV3-23 | BCRSignalingPathway |
| IGHV3-30 | BCRSignalingPathway |
| IGHV3-30-3 | BCRSignalingPathway |
| IGHV3-30-5 | BCRSignalingPathway |
| IGHV3-33 | BCRSignalingPathway |
| IGHV3-35 | BCRSignalingPathway |
| IGHV3-38 | BCRSignalingPathway |

| IGHV3-43 | BCRSignalingPathway |
| --- | --- |
| IGHV3-48 | BCRSignalingPathway |
| IGHV3-49 | BCRSignalingPathway |
| IGHV3-53 | BCRSignalingPathway |
| IGHV3-64 | BCRSignalingPathway |
| IGHV3-66 | BCRSignalingPathway |
| IGHV3-7 | BCRSignalingPathway |
| IGHV3-72 | BCRSignalingPathway |
| IGHV3-73 | BCRSignalingPathway |
| IGHV3-74 | BCRSignalingPathway |
| IGHV3-9 | BCRSignalingPathway |
| IGHV3-D | BCRSignalingPathway |
| IGHV3-H | BCRSignalingPathway |
| IGHV4-28 | BCRSignalingPathway |
| IGHV4-30-1 | BCRSignalingPathway |
| IGHV4-30-2 | BCRSignalingPathway |
| IGHV4-30-4 | BCRSignalingPathway |
| IGHV4-31 | BCRSignalingPathway |
| IGHV4-34 | BCRSignalingPathway |
| IGHV4-39 | BCRSignalingPathway |
| IGHV4-4 | BCRSignalingPathway |
| IGHV4-59 | BCRSignalingPathway |
| IGHV4-61 | BCRSignalingPathway |
| IGHV4-B | BCRSignalingPathway |
| IGHV5-51 | BCRSignalingPathway |
| IGHV5-A | BCRSignalingPathway |
| IGHV6-1 | BCRSignalingPathway |
| IGHV7-4-1 | BCRSignalingPathway |
| IGHV7-81 | BCRSignalingPathway |
| IGK@ | BCRSignalingPathway |
| IGKC | BCRSignalingPathway |
| IGKDEL | BCRSignalingPathway |
| IGKJ@ | BCRSignalingPathway |
| IGKJ1 | BCRSignalingPathway |
| IGKJ2 | BCRSignalingPathway |
| IGKJ3 | BCRSignalingPathway |
| IGKJ4 | BCRSignalingPathway |
| IGKJ5 | BCRSignalingPathway |
| IGKV@ | BCRSignalingPathway |
| IGKV1-12 | BCRSignalingPathway |
| IGKV1-13 | BCRSignalingPathway |
| IGKV1-16 | BCRSignalingPathway |
| IGKV1-17 | BCRSignalingPathway |
| IGKV1-27 | BCRSignalingPathway |
| IGKV1-33 | BCRSignalingPathway |
| IGKV1-37 | BCRSignalingPathway |
| IGKV1-39 | BCRSignalingPathway |
| IGKV1-5 | BCRSignalingPathway |
| IGKV1-6 | BCRSignalingPathway |
| IGKV1-8 | BCRSignalingPathway |
| IGKV1-9 | BCRSignalingPathway |
| IGKV1D-12 | BCRSignalingPathway |

| IGKV1D-13 | BCRSignalingPathway |
| --- | --- |
| IGKV1D-16 | BCRSignalingPathway |
| IGKV1D-17 | BCRSignalingPathway |
| IGKV1D-33 | BCRSignalingPathway |
| IGKV1D-37 | BCRSignalingPathway |
| IGKV1D-39 | BCRSignalingPathway |
| IGKV1D-42 | BCRSignalingPathway |
| IGKV1D-43 | BCRSignalingPathway |
| IGKV1D-8 | BCRSignalingPathway |
| IGKV2-24 | BCRSignalingPathway |
| IGKV2-28 | BCRSignalingPathway |
| IGKV2-30 | BCRSignalingPathway |
| IGKV2-40 | BCRSignalingPathway |
| IGKV2D-24 | BCRSignalingPathway |
| IGKV2D-28 | BCRSignalingPathway |
| IGKV2D-29 | BCRSignalingPathway |
| IGKV2D-30 | BCRSignalingPathway |
| IGKV2D-40 | BCRSignalingPathway |
| IGKV3-11 | BCRSignalingPathway |
| IGKV3-15 | BCRSignalingPathway |
| IGKV3-20 | BCRSignalingPathway |
| IGKV3-7 | BCRSignalingPathway |
| IGKV3D-11 | BCRSignalingPathway |
| IGKV3D-15 | BCRSignalingPathway |
| IGKV3D-20 | BCRSignalingPathway |
| IGKV3D-7 | BCRSignalingPathway |
| IGKV4-1 | BCRSignalingPathway |
| IGKV5-2 | BCRSignalingPathway |
| IGKV6-21 | BCRSignalingPathway |
| IGKV6D-21 | BCRSignalingPathway |
| IGKV6D-41 | BCRSignalingPathway |
| IGL@ | BCRSignalingPathway |
| IGLC@ | BCRSignalingPathway |
| IGLC1 | BCRSignalingPathway |
| IGLC2 | BCRSignalingPathway |
| IGLC3 | BCRSignalingPathway |
| IGLC6 | BCRSignalingPathway |
| IGLC7 | BCRSignalingPathway |
| IGLJ@ | BCRSignalingPathway |
| IGLJ1 | BCRSignalingPathway |
| IGLJ2 | BCRSignalingPathway |
| IGLJ3 | BCRSignalingPathway |
| IGLJ4 | BCRSignalingPathway |
| IGLJ5 | BCRSignalingPathway |
| IGLJ6 | BCRSignalingPathway |
| IGLJ7 | BCRSignalingPathway |
| IGLV@ | BCRSignalingPathway |
| IGLV1-36 | BCRSignalingPathway |
| IGLV1-40 | BCRSignalingPathway |
| IGLV1-44 | BCRSignalingPathway |
| IGLV1-47 | BCRSignalingPathway |
| IGLV1-50 | BCRSignalingPathway |

| IGLV1-51 | BCRSignalingPathway |
| --- | --- |
| IGLV10-54 | BCRSignalingPathway |
| IGLV11-55 | BCRSignalingPathway |
| IGLV2-11 | BCRSignalingPathway |
| IGLV2-14 | BCRSignalingPathway |
| IGLV2-18 | BCRSignalingPathway |
| IGLV2-23 | BCRSignalingPathway |
| IGLV2-33 | BCRSignalingPathway |
| IGLV2-8 | BCRSignalingPathway |
| IGLV3-1 | BCRSignalingPathway |
| IGLV3-10 | BCRSignalingPathway |
| IGLV3-12 | BCRSignalingPathway |
| IGLV3-16 | BCRSignalingPathway |
| IGLV3-19 | BCRSignalingPathway |
| IGLV3-21 | BCRSignalingPathway |
| IGLV3-22 | BCRSignalingPathway |
| IGLV3-25 | BCRSignalingPathway |
| IGLV3-27 | BCRSignalingPathway |
| IGLV3-32 | BCRSignalingPathway |
| IGLV3-9 | BCRSignalingPathway |
| IGLV4-3 | BCRSignalingPathway |
| IGLV4-60 | BCRSignalingPathway |
| IGLV4-69 | BCRSignalingPathway |
| IGLV5-37 | BCRSignalingPathway |
| IGLV5-39 | BCRSignalingPathway |
| IGLV5-45 | BCRSignalingPathway |
| IGLV5-48 | BCRSignalingPathway |
| IGLV5-52 | BCRSignalingPathway |
| IGLV6-57 | BCRSignalingPathway |
| IGLV7-43 | BCRSignalingPathway |
| IGLV7-46 | BCRSignalingPathway |
| IGLV8-61 | BCRSignalingPathway |
| IGLV9-49 | BCRSignalingPathway |
| C3 | Chemokines |
| C5 | Chemokines |
| CAMP | Chemokines |
| CCL1 | Chemokines |
| CCL11 | Chemokines |
| CCL13 | Chemokines |
| CCL14 | Chemokines |
| CCL14-CCL15 | Chemokines |
| CCL15 | Chemokines |
| CCL16 | Chemokines |
| CCL17 | Chemokines |
| CCL18 | Chemokines |
| CCL19 | Chemokines |
| CCL2 | Chemokines |
| CCL20 | Chemokines |
| CCL21 | Chemokines |
| CCL22 | Chemokines |
| CCL23 | Chemokines |
| CCL24 | Chemokines |

| CCL25 | Chemokines |
| --- | --- |
| CCL26 | Chemokines |
| CCL27 | Chemokines |
| CCL28 | Chemokines |
| CCL3 | Chemokines |
| CCL3L1 | Chemokines |
| CCL3L2 | Chemokines |
| CCL3L3 | Chemokines |
| CCL4 | Chemokines |
| CCL4L1 | Chemokines |
| CCL4L2 | Chemokines |
| CCL5 | Chemokines |
| CCL7 | Chemokines |
| CCL8 | Chemokines |
| CKLF | Chemokines |
| CMA1 | Chemokines |
| CTSG | Chemokines |
| CX3CL1 | Chemokines |
| CXCL1 | Chemokines |
| CXCL10 | Chemokines |
| CXCL11 | Chemokines |
| CXCL12 | Chemokines |
| CXCL13 | Chemokines |
| CXCL14 | Chemokines |
| CXCL16 | Chemokines |
| CXCL17 | Chemokines |
| CXCL2 | Chemokines |
| CXCL3 | Chemokines |
| CXCL5 | Chemokines |
| CXCL6 | Chemokines |
| CXCL9 | Chemokines |
| CYR61 | Chemokines |
| DEFA1 | Chemokines |
| DEFA3 | Chemokines |
| DEFA5 | Chemokines |
| DEFB1 | Chemokines |
| DEFB103A | Chemokines |
| DEFB104A | Chemokines |
| DEFB4 | Chemokines |
| EDN1 | Chemokines |
| EDN2 | Chemokines |
| EDN3 | Chemokines |
| FGF10 | Chemokines |
| FGF2 | Chemokines |
| HTN3 | Chemokines |
| IL8 | Chemokines |
| LECT2 | Chemokines |
| PF4 | Chemokines |
| PF4V1 | Chemokines |
| PLAU | Chemokines |
| PPBP | Chemokines |
| PPBPL1 | Chemokines |

| PROK2 | Chemokines |
| --- | --- |
| RNASE2 | Chemokines |
| SAA1 | Chemokines |
| SAA2 | Chemokines |
| SBDS | Chemokines |
| SEMA3A | Chemokines |
| SEMA3B | Chemokines |
| SEMA3C | Chemokines |
| SEMA3D | Chemokines |
| SEMA3E | Chemokines |
| SEMA3F | Chemokines |
| SEMA3G | Chemokines |
| SEMA4A | Chemokines |
| SEMA4B | Chemokines |
| SEMA4C | Chemokines |
| SEMA4D | Chemokines |
| SEMA4F | Chemokines |
| SEMA4G | Chemokines |
| SEMA5A | Chemokines |
| SEMA5B | Chemokines |
| SEMA6A | Chemokines |
| SEMA6B | Chemokines |
| SEMA6C | Chemokines |
| SEMA6D | Chemokines |
| SEMA7A | Chemokines |
| SLIT1 | Chemokines |
| SLIT2 | Chemokines |
| TNC | Chemokines |
| TYMP | Chemokines |
| XCL1 | Chemokines |
| XCL2 | Chemokines |
| C5AR1 | Chemokine_Receptors |
| CCBP2 | Chemokine_Receptors |
| CCR1 | Chemokine_Receptors |
| CCR10 | Chemokine_Receptors |
| CCR3 | Chemokine_Receptors |
| CCR4 | Chemokine_Receptors |
| CCR5 | Chemokine_Receptors |
| CCR6 | Chemokine_Receptors |
| CCR7 | Chemokine_Receptors |
| CCR8 | Chemokine_Receptors |
| CCR9 | Chemokine_Receptors |
| CCRL1 | Chemokine_Receptors |
| CCRL2 | Chemokine_Receptors |
| CMKLR1 | Chemokine_Receptors |
| CX3CR1 | Chemokine_Receptors |
| CXCR3 | Chemokine_Receptors |
| CXCR4 | Chemokine_Receptors |
| CXCR5 | Chemokine_Receptors |
| CXCR6 | Chemokine_Receptors |
| CXCR7 | Chemokine_Receptors |
| CYSLTR1 | Chemokine_Receptors |

| CYSLTR2 | Chemokine_Receptors |
| --- | --- |
| DARC | Chemokine_Receptors |
| EDNRA | Chemokine_Receptors |
| EDNRB | Chemokine_Receptors |
| FPR1 | Chemokine_Receptors |
| FPR2 | Chemokine_Receptors |
| FPR2 | Chemokine_Receptors |
| GPR17 | Chemokine_Receptors |
| GPR32 | Chemokine_Receptors |
| GPR33 | Chemokine_Receptors |
| GPR44 | Chemokine_Receptors |
| GPR77 | Chemokine_Receptors |
| IL8RA | Chemokine_Receptors |
| IL8RB | Chemokine_Receptors |
| LTB4R | Chemokine_Receptors |
| LTB4R2 | Chemokine_Receptors |
| PLAUR | Chemokine_Receptors |
| PLXNA1 | Chemokine_Receptors |
| PLXNA2 | Chemokine_Receptors |
| PLXNA3 | Chemokine_Receptors |
| PLXNA4 | Chemokine_Receptors |
| PLXNB1 | Chemokine_Receptors |
| PLXNB2 | Chemokine_Receptors |
| PLXNB3 | Chemokine_Receptors |
| PLXNC1 | Chemokine_Receptors |
| PLXND1 | Chemokine_Receptors |
| PTAFR | Chemokine_Receptors |
| ROBO1 | Chemokine_Receptors |
| ROBO2 | Chemokine_Receptors |
| ROBO3 | Chemokine_Receptors |
| RXFP3 | Chemokine_Receptors |
| XCR1 | Chemokine_Receptors |
| ADIPOQ | Cytokines |
| ADM | Cytokines |
| ADM2 | Cytokines |
| AGRP | Cytokines |
| AGT | Cytokines |
| AMBN | Cytokines |
| AMELX | Cytokines |
| AMH | Cytokines |
| ANGPTL5 | Cytokines |
| ANGPTL7 | Cytokines |
| APLN | Cytokines |
| AREG | Cytokines |
| ARMET | Cytokines |
| ARMETL1 | Cytokines |
| ARTN | Cytokines |
| AVP | Cytokines |
| AZU1 | Cytokines |
| BDNF | Cytokines |
| BMP1 | Cytokines |
| BMP10 | Cytokines |

| BMP15 | Cytokines |
| --- | --- |
| BMP2 | Cytokines |
| BMP3 | Cytokines |
| BMP4 | Cytokines |
| BMP5 | Cytokines |
| BMP6 | Cytokines |
| BMP7 | Cytokines |
| BMP8A | Cytokines |
| BMP8B | Cytokines |
| BTC | Cytokines |
| C19orf10 | Cytokines |
| C3 | Cytokines |
| C5 | Cytokines |
| CALCA | Cytokines |
| CALCB | Cytokines |
| CAMP | Cytokines |
| CAT | Cytokines |
| CCK | Cytokines |
| CCL1 | Cytokines |
| CCL11 | Cytokines |
| CCL13 | Cytokines |
| CCL14 | Cytokines |
| CCL14-CCL15 | Cytokines |
| CCL15 | Cytokines |
| CCL16 | Cytokines |
| CCL17 | Cytokines |
| CCL18 | Cytokines |
| CCL19 | Cytokines |
| CCL2 | Cytokines |
| CCL20 | Cytokines |
| CCL21 | Cytokines |
| CCL22 | Cytokines |
| CCL23 | Cytokines |
| CCL24 | Cytokines |
| CCL25 | Cytokines |
| CCL26 | Cytokines |
| CCL27 | Cytokines |
| CCL28 | Cytokines |
| CCL3 | Cytokines |
| CCL3L1 | Cytokines |
| CCL3L2 | Cytokines |
| CCL3L3 | Cytokines |
| CCL4 | Cytokines |
| CCL4L1 | Cytokines |
| CCL4L2 | Cytokines |
| CCL5 | Cytokines |
| CCL7 | Cytokines |
| CCL8 | Cytokines |
| CD320 | Cytokines |
| CD40LG | Cytokines |
| CD70 | Cytokines |
| CECR1 | Cytokines |

| CER1 | Cytokines |
| --- | --- |
| CGA | Cytokines |
| CGB | Cytokines |
| CGB1 | Cytokines |
| CGB2 | Cytokines |
| CGB5 | Cytokines |
| CGB7 | Cytokines |
| CGB8 | Cytokines |
| CHGA | Cytokines |
| CHGB | Cytokines |
| CKLF | Cytokines |
| CLCF1 | Cytokines |
| CLEC11A | Cytokines |
| CMA1 | Cytokines |
| CMTM1 | Cytokines |
| CMTM2 | Cytokines |
| CMTM3 | Cytokines |
| CMTM4 | Cytokines |
| CMTM5 | Cytokines |
| CMTM6 | Cytokines |
| CMTM7 | Cytokines |
| CMTM8 | Cytokines |
| CNTF | Cytokines |
| CORT | Cytokines |
| CRH | Cytokines |
| CSF1 | Cytokines |
| CSF2 | Cytokines |
| CSF3 | Cytokines |
| CSH1 | Cytokines |
| CSH2 | Cytokines |
| CSHL1 | Cytokines |
| CSPG5 | Cytokines |
| CTF1 | Cytokines |
| CTGF | Cytokines |
| CTSG | Cytokines |
| CX3CL1 | Cytokines |
| CXCL1 | Cytokines |
| CXCL10 | Cytokines |
| CXCL11 | Cytokines |
| CXCL12 | Cytokines |
| CXCL13 | Cytokines |
| CXCL14 | Cytokines |
| CXCL16 | Cytokines |
| CXCL17 | Cytokines |
| CXCL2 | Cytokines |
| CXCL3 | Cytokines |
| CXCL5 | Cytokines |
| CXCL6 | Cytokines |
| CXCL9 | Cytokines |
| CYR61 | Cytokines |
| DEFA1 | Cytokines |
| DEFA3 | Cytokines |

| DEFA5 | Cytokines |
| --- | --- |
| DEFB1 | Cytokines |
| DEFB103A | Cytokines |
| DEFB104A | Cytokines |
| DEFB4 | Cytokines |
| DKK1 | Cytokines |
| EBI3 | Cytokines |
| EDN1 | Cytokines |
| EDN2 | Cytokines |
| EDN3 | Cytokines |
| EGF | Cytokines |
| EPGN | Cytokines |
| EPO | Cytokines |
| EREG | Cytokines |
| ESM1 | Cytokines |
| FAM3B | Cytokines |
| FAM3C | Cytokines |
| FAM3D | Cytokines |
| FASLG | Cytokines |
| FGF1 | Cytokines |
| FGF10 | Cytokines |
| FGF11 | Cytokines |
| FGF12 | Cytokines |
| FGF13 | Cytokines |
| FGF14 | Cytokines |
| FGF16 | Cytokines |
| FGF17 | Cytokines |
| FGF18 | Cytokines |
| FGF19 | Cytokines |
| FGF2 | Cytokines |
| FGF20 | Cytokines |
| FGF21 | Cytokines |
| FGF22 | Cytokines |
| FGF23 | Cytokines |
| FGF3 | Cytokines |
| FGF4 | Cytokines |
| FGF5 | Cytokines |
| FGF6 | Cytokines |
| FGF7 | Cytokines |
| FGF8 | Cytokines |
| FGF9 | Cytokines |
| FIGF | Cytokines |
| FIGNL2 | Cytokines |
| FLT3LG | Cytokines |
| FSHB | Cytokines |
| GAL | Cytokines |
| GALP | Cytokines |
| GAST | Cytokines |
| GCG | Cytokines |
| GDF1 | Cytokines |
| GDF10 | Cytokines |
| GDF11 | Cytokines |

| GDF15 | Cytokines |
| --- | --- |
| GDF2 | Cytokines |
| GDF3 | Cytokines |
| GDF5 | Cytokines |
| GDF6 | Cytokines |
| GDF7 | Cytokines |
| GDF9 | Cytokines |
| GDNF | Cytokines |
| GH1 | Cytokines |
| GH2 | Cytokines |
| GHRH | Cytokines |
| GHRL | Cytokines |
| GIP | Cytokines |
| GKN1 | Cytokines |
| GMFB | Cytokines |
| GMFG | Cytokines |
| GNRH1 | Cytokines |
| GNRH2 | Cytokines |
| GPHA2 | Cytokines |
| GPHB5 | Cytokines |
| GPI | Cytokines |
| GREM1 | Cytokines |
| GREM2 | Cytokines |
| GRN | Cytokines |
| GRP | Cytokines |
| GUCA2A | Cytokines |
| HAMP | Cytokines |
| HBEGF | Cytokines |
| HDGF | Cytokines |
| HDGFRP3 | Cytokines |
| HGF | Cytokines |
| HTN3 | Cytokines |
| IAPP | Cytokines |
| IFNA1 | Cytokines |
| IFNA10 | Cytokines |
| IFNA13 | Cytokines |
| IFNA14 | Cytokines |
| IFNA16 | Cytokines |
| IFNA17 | Cytokines |
| IFNA2 | Cytokines |
| IFNA21 | Cytokines |
| IFNA4 | Cytokines |
| IFNA5 | Cytokines |
| IFNA6 | Cytokines |
| IFNA7 | Cytokines |
| IFNA8 | Cytokines |
| IFNB1 | Cytokines |
| IFNE | Cytokines |
| IFNG | Cytokines |
| IFNK | Cytokines |
| IFNW1 | Cytokines |
| IGF1 | Cytokines |

| IGF2 | Cytokines |
| --- | --- |
| IL10 | Cytokines |
| IL11 | Cytokines |
| IL12A | Cytokines |
| IL12B | Cytokines |
| IL13 | Cytokines |
| IL15 | Cytokines |
| IL16 | Cytokines |
| IL17A | Cytokines |
| IL17B | Cytokines |
| IL17C | Cytokines |
| IL17D | Cytokines |
| IL17F | Cytokines |
| IL18 | Cytokines |
| IL19 | Cytokines |
| IL1A | Cytokines |
| IL1B | Cytokines |
| IL1F10 | Cytokines |
| IL1F5 | Cytokines |
| IL1F6 | Cytokines |
| IL1F7 | Cytokines |
| IL1F8 | Cytokines |
| IL1F9 | Cytokines |
| IL1RN | Cytokines |
| IL2 | Cytokines |
| IL20 | Cytokines |
| IL21 | Cytokines |
| IL22 | Cytokines |
| IL23A | Cytokines |
| IL24 | Cytokines |
| IL25 | Cytokines |
| IL26 | Cytokines |
| IL27 | Cytokines |
| IL28A | Cytokines |
| IL28B | Cytokines |
| IL29 | Cytokines |
| IL3 | Cytokines |
| IL31 | Cytokines |
| IL32 | Cytokines |
| IL33 | Cytokines |
| IL34 | Cytokines |
| IL4 | Cytokines |
| IL5 | Cytokines |
| IL6 | Cytokines |
| IL6ST | Cytokines |
| IL7 | Cytokines |
| IL8 | Cytokines |
| IL9 | Cytokines |
| INHA | Cytokines |
| INHBA | Cytokines |
| INHBB | Cytokines |
| INHBC | Cytokines |

| INHBE | Cytokines |
| --- | --- |
| INS | Cytokines |
| INS-IGF2 | Cytokines |
| INSL3 | Cytokines |
| INSL4 | Cytokines |
| INSL5 | Cytokines |
| INSL6 | Cytokines |
| JAG1 | Cytokines |
| JAG2 | Cytokines |
| KGFLP1 | Cytokines |
| KGFLP2 | Cytokines |
| KITLG | Cytokines |
| KL | Cytokines |
| LACRT | Cytokines |
| LECT2 | Cytokines |
| LEFTY1 | Cytokines |
| LEFTY2 | Cytokines |
| LEP | Cytokines |
| LHB | Cytokines |
| LIF | Cytokines |
| LRSAM1 | Cytokines |
| LTA | Cytokines |
| LTB | Cytokines |
| LTBP1 | Cytokines |
| LTBP2 | Cytokines |
| LTBP3 | Cytokines |
| LTBP4 | Cytokines |
| MDK | Cytokines |
| MIA | Cytokines |
| MIF | Cytokines |
| MLN | Cytokines |
| MSTN | Cytokines |
| NAMPT | Cytokines |
| NDP | Cytokines |
| NENF | Cytokines |
| NGF | Cytokines |
| NMB | Cytokines |
| NODAL | Cytokines |
| NOV | Cytokines |
| NPFF | Cytokines |
| NPPA | Cytokines |
| NPPB | Cytokines |
| NPPC | Cytokines |
| NPY | Cytokines |
| NRG1 | Cytokines |
| NRG2 | Cytokines |
| NRG3 | Cytokines |
| NRG4 | Cytokines |
| NRTN | Cytokines |
| NTF3 | Cytokines |
| NTF4 | Cytokines |
| NTS | Cytokines |

| NUDT6 | Cytokines |
| --- | --- |
| OGN | Cytokines |
| OSGIN1 | Cytokines |
| OSM | Cytokines |
| OSTN | Cytokines |
| OXT | Cytokines |
| P11 | Cytokines |
| PDGFA | Cytokines |
| PDGFB | Cytokines |
| PDGFC | Cytokines |
| PDGFD | Cytokines |
| PDGFRA | Cytokines |
| PDGFRB | Cytokines |
| PDGFRL | Cytokines |
| PDYN | Cytokines |
| PENK | Cytokines |
| PF4 | Cytokines |
| PF4V1 | Cytokines |
| PGF | Cytokines |
| PLAU | Cytokines |
| PMCH | Cytokines |
| PNOC | Cytokines |
| POMC | Cytokines |
| PPBP | Cytokines |
| PPBPL1 | Cytokines |
| PPBPL2 | Cytokines |
| PPY | Cytokines |
| PRL | Cytokines |
| PRLH | Cytokines |
| PROK1 | Cytokines |
| PROK2 | Cytokines |
| PSPN | Cytokines |
| PTH | Cytokines |
| PTH2 | Cytokines |
| PTHLH | Cytokines |
| PTN | Cytokines |
| PYY | Cytokines |
| QRFP | Cytokines |
| RABEP1 | Cytokines |
| RABEP2 | Cytokines |
| REG1A | Cytokines |
| RETN | Cytokines |
| RETNLB | Cytokines |
| RLN1 | Cytokines |
| RLN2 | Cytokines |
| RLN3 | Cytokines |
| RNASE2 | Cytokines |
| S100A6 | Cytokines |
| SAA1 | Cytokines |
| SAA2 | Cytokines |
| SBDS | Cytokines |
| SCG2 | Cytokines |

| SCGB3A1 | Cytokines |
| --- | --- |
| SCT | Cytokines |
| SCYE1 | Cytokines |
| SECTM1 | Cytokines |
| SEMA3A | Cytokines |
| SEMA3B | Cytokines |
| SEMA3C | Cytokines |
| SEMA3D | Cytokines |
| SEMA3E | Cytokines |
| SEMA3F | Cytokines |
| SEMA3G | Cytokines |
| SEMA4A | Cytokines |
| SEMA4B | Cytokines |
| SEMA4C | Cytokines |
| SEMA4D | Cytokines |
| SEMA4F | Cytokines |
| SEMA4G | Cytokines |
| SEMA5A | Cytokines |
| SEMA5B | Cytokines |
| SEMA6A | Cytokines |
| SEMA6B | Cytokines |
| SEMA6C | Cytokines |
| SEMA6D | Cytokines |
| SEMA7A | Cytokines |
| SLIT1 | Cytokines |
| SLIT2 | Cytokines |
| SLURP1 | Cytokines |
| SPP1 | Cytokines |
| SST | Cytokines |
| STC1 | Cytokines |
| STC2 | Cytokines |
| TAC1 | Cytokines |
| TDGF1 | Cytokines |
| TDGF3 | Cytokines |
| TG | Cytokines |
| TGFA | Cytokines |
| TGFB1 | Cytokines |
| TGFB2 | Cytokines |
| TGFB3 | Cytokines |
| THPO | Cytokines |
| TNC | Cytokines |
| TNF | Cytokines |
| TNFRSF11B | Cytokines |
| TNFSF10 | Cytokines |
| TNFSF11 | Cytokines |
| TNFSF12 | Cytokines |
| TNFSF13 | Cytokines |
| TNFSF13B | Cytokines |
| TNFSF14 | Cytokines |
| TNFSF15 | Cytokines |
| TNFSF18 | Cytokines |
| TNFSF4 | Cytokines |

| TNFSF8 | Cytokines |
| --- | --- |
| TNFSF9 | Cytokines |
| TOR2A | Cytokines |
| TRH | Cytokines |
| TSHB | Cytokines |
| TSLP | Cytokines |
| TXLNA | Cytokines |
| TYMP | Cytokines |
| UCN | Cytokines |
| UCN2 | Cytokines |
| UCN3 | Cytokines |
| UTS2 | Cytokines |
| UTS2D | Cytokines |
| VEGFA | Cytokines |
| VEGFB | Cytokines |
| VEGFC | Cytokines |
| VGF | Cytokines |
| VIP | Cytokines |
| XCL1 | Cytokines |
| XCL2 | Cytokines |
| ACVR1B | Cytokine_Receptors |
| ACVR1C | Cytokine_Receptors |
| ACVR2A | Cytokine_Receptors |
| ACVR2B | Cytokine_Receptors |
| ACVRL1 | Cytokine_Receptors |
| ADCYAP1R1 | Cytokine_Receptors |
| ADIPOR1 | Cytokine_Receptors |
| ADIPOR2 | Cytokine_Receptors |
| ADRB1 | Cytokine_Receptors |
| ADRB2 | Cytokine_Receptors |
| AGTR1 | Cytokine_Receptors |
| AGTR2 | Cytokine_Receptors |
| AMHR2 | Cytokine_Receptors |
| ANGPT1 | Cytokine_Receptors |
| ANGPT4 | Cytokine_Receptors |
| ANGPTL1 | Cytokine_Receptors |
| ANGPTL2 | Cytokine_Receptors |
| ANGPTL3 | Cytokine_Receptors |
| ANGPTL4 | Cytokine_Receptors |
| ANGPTL6 | Cytokine_Receptors |
| APLNR | Cytokine_Receptors |
| AR | Cytokine_Receptors |
| AVPR1A | Cytokine_Receptors |
| AVPR1B | Cytokine_Receptors |
| AVPR2 | Cytokine_Receptors |
| BMPR1A | Cytokine_Receptors |
| BMPR1B | Cytokine_Receptors |
| BMPR2 | Cytokine_Receptors |
| BRD8 | Cytokine_Receptors |
| C3AR1 | Cytokine_Receptors |
| C5AR1 | Cytokine_Receptors |
| CALCR | Cytokine_Receptors |

| CALCRL | Cytokine_Receptors |
| --- | --- |
| CCBP2 | Cytokine_Receptors |
| CCR1 | Cytokine_Receptors |
| CCR10 | Cytokine_Receptors |
| CCR3 | Cytokine_Receptors |
| CCR4 | Cytokine_Receptors |
| CCR5 | Cytokine_Receptors |
| CCR6 | Cytokine_Receptors |
| CCR7 | Cytokine_Receptors |
| CCR8 | Cytokine_Receptors |
| CCR9 | Cytokine_Receptors |
| CCRL1 | Cytokine_Receptors |
| CCRL2 | Cytokine_Receptors |
| CD40 | Cytokine_Receptors |
| CMKLR1 | Cytokine_Receptors |
| CNTFR | Cytokine_Receptors |
| CRHR1 | Cytokine_Receptors |
| CRHR2 | Cytokine_Receptors |
| CRIM1 | Cytokine_Receptors |
| CRLF1 | Cytokine_Receptors |
| CRLF2 | Cytokine_Receptors |
| CRLF3 | Cytokine_Receptors |
| CSF1R | Cytokine_Receptors |
| CSF2RA | Cytokine_Receptors |
| CSF2RB | Cytokine_Receptors |
| CSF3R | Cytokine_Receptors |
| CX3CR1 | Cytokine_Receptors |
| CXCR3 | Cytokine_Receptors |
| CXCR4 | Cytokine_Receptors |
| CXCR5 | Cytokine_Receptors |
| CXCR6 | Cytokine_Receptors |
| CXCR7 | Cytokine_Receptors |
| CYSLTR1 | Cytokine_Receptors |
| CYSLTR2 | Cytokine_Receptors |
| DARC | Cytokine_Receptors |
| EDNRA | Cytokine_Receptors |
| EDNRB | Cytokine_Receptors |
| EGFR | Cytokine_Receptors |
| ENG | Cytokine_Receptors |
| EPOR | Cytokine_Receptors |
| ESR1 | Cytokine_Receptors |
| ESR2 | Cytokine_Receptors |
| ESRRA | Cytokine_Receptors |
| ESRRB | Cytokine_Receptors |
| ESRRG | Cytokine_Receptors |
| FGFR1 | Cytokine_Receptors |
| FGFR2 | Cytokine_Receptors |
| FGFR3 | Cytokine_Receptors |
| FGFR4 | Cytokine_Receptors |
| FGFRL1 | Cytokine_Receptors |
| FLT1 | Cytokine_Receptors |
| FLT3 | Cytokine_Receptors |

| FLT4 | Cytokine_Receptors |
| --- | --- |
| FPR1 | Cytokine_Receptors |
| FPR2 | Cytokine_Receptors |
| FPR2 | Cytokine_Receptors |
| FSHR | Cytokine_Receptors |
| GALR2 | Cytokine_Receptors |
| GALR3 | Cytokine_Receptors |
| GCGR | Cytokine_Receptors |
| GHR | Cytokine_Receptors |
| GHRHR | Cytokine_Receptors |
| GHSR | Cytokine_Receptors |
| GIPR | Cytokine_Receptors |
| GLP1R | Cytokine_Receptors |
| GLP2R | Cytokine_Receptors |
| GNRHR | Cytokine_Receptors |
| GPER | Cytokine_Receptors |
| GPR17 | Cytokine_Receptors |
| GPR32 | Cytokine_Receptors |
| GPR33 | Cytokine_Receptors |
| GPR44 | Cytokine_Receptors |
| GPR77 | Cytokine_Receptors |
| HNF4A | Cytokine_Receptors |
| HNF4G | Cytokine_Receptors |
| HTR3A | Cytokine_Receptors |
| HTR3B | Cytokine_Receptors |
| HTR3C | Cytokine_Receptors |
| HTR3D | Cytokine_Receptors |
| HTR3E | Cytokine_Receptors |
| IFNAR1 | Cytokine_Receptors |
| IFNAR2 | Cytokine_Receptors |
| IFNGR1 | Cytokine_Receptors |
| IFNGR2 | Cytokine_Receptors |
| IGF1R | Cytokine_Receptors |
| IGF2R | Cytokine_Receptors |
| IL10RA | Cytokine_Receptors |
| IL10RB | Cytokine_Receptors |
| IL11RA | Cytokine_Receptors |
| IL11RB | Cytokine_Receptors |
| IL12RB1 | Cytokine_Receptors |
| IL12RB2 | Cytokine_Receptors |
| IL13RA1 | Cytokine_Receptors |
| IL13RA2 | Cytokine_Receptors |
| IL15RA | Cytokine_Receptors |
| IL15RB | Cytokine_Receptors |
| IL17RA | Cytokine_Receptors |
| IL17RB | Cytokine_Receptors |
| IL17RC | Cytokine_Receptors |
| IL17RD | Cytokine_Receptors |
| IL17RE | Cytokine_Receptors |
| IL18R1 | Cytokine_Receptors |
| IL18RAP | Cytokine_Receptors |
| IL1R1 | Cytokine_Receptors |

| IL1R2 | Cytokine_Receptors |
| --- | --- |
| IL1RAP | Cytokine_Receptors |
| IL1RL1 | Cytokine_Receptors |
| IL1RL2 | Cytokine_Receptors |
| IL20RA | Cytokine_Receptors |
| IL20RB | Cytokine_Receptors |
| IL21R | Cytokine_Receptors |
| IL22RA1 | Cytokine_Receptors |
| IL22RA2 | Cytokine_Receptors |
| IL23R | Cytokine_Receptors |
| IL27RA | Cytokine_Receptors |
| IL28RA | Cytokine_Receptors |
| IL2RA | Cytokine_Receptors |
| IL2RB | Cytokine_Receptors |
| IL2RG | Cytokine_Receptors |
| IL31RA | Cytokine_Receptors |
| IL3RA | Cytokine_Receptors |
| IL4R | Cytokine_Receptors |
| IL5RA | Cytokine_Receptors |
| IL6R | Cytokine_Receptors |
| IL7R | Cytokine_Receptors |
| IL8RA | Cytokine_Receptors |
| IL8RB | Cytokine_Receptors |
| IL9R | Cytokine_Receptors |
| INSR | Cytokine_Receptors |
| KDR | Cytokine_Receptors |
| LEPR | Cytokine_Receptors |
| LGR4 | Cytokine_Receptors |
| LGR5 | Cytokine_Receptors |
| LGR6 | Cytokine_Receptors |
| LHCGR | Cytokine_Receptors |
| LIFR | Cytokine_Receptors |
| LTB4R | Cytokine_Receptors |
| LTB4R2 | Cytokine_Receptors |
| LTBR | Cytokine_Receptors |
| MC1R | Cytokine_Receptors |
| MC2R | Cytokine_Receptors |
| MC3R | Cytokine_Receptors |
| MC4R | Cytokine_Receptors |
| MCHR1 | Cytokine_Receptors |
| MCHR2 | Cytokine_Receptors |
| MET | Cytokine_Receptors |
| MLNR | Cytokine_Receptors |
| MPL | Cytokine_Receptors |
| MTNR1A | Cytokine_Receptors |
| MTNR1B | Cytokine_Receptors |
| NGFR | Cytokine_Receptors |
| NMBR | Cytokine_Receptors |
| NPR1 | Cytokine_Receptors |
| NPR3 | Cytokine_Receptors |
| NR0B1 | Cytokine_Receptors |
| NR0B2 | Cytokine_Receptors |

| NR1D1 | Cytokine_Receptors |
| --- | --- |
| NR1D2 | Cytokine_Receptors |
| NR1H2 | Cytokine_Receptors |
| NR1H3 | Cytokine_Receptors |
| NR1H4 | Cytokine_Receptors |
| NR1I2 | Cytokine_Receptors |
| NR1I3 | Cytokine_Receptors |
| NR2C1 | Cytokine_Receptors |
| NR2C2 | Cytokine_Receptors |
| NR2E1 | Cytokine_Receptors |
| NR2E3 | Cytokine_Receptors |
| NR2F1 | Cytokine_Receptors |
| NR2F2 | Cytokine_Receptors |
| NR2F6 | Cytokine_Receptors |
| NR3C1 | Cytokine_Receptors |
| NR3C2 | Cytokine_Receptors |
| NR4A1 | Cytokine_Receptors |
| NR4A2 | Cytokine_Receptors |
| NR4A3 | Cytokine_Receptors |
| NR5A1 | Cytokine_Receptors |
| NR5A2 | Cytokine_Receptors |
| NR6A1 | Cytokine_Receptors |
| NRP1 | Cytokine_Receptors |
| NRP2 | Cytokine_Receptors |
| OGFR | Cytokine_Receptors |
| OPRD1 | Cytokine_Receptors |
| OPRK1 | Cytokine_Receptors |
| OPRL1 | Cytokine_Receptors |
| OPRM1 | Cytokine_Receptors |
| OSMR | Cytokine_Receptors |
| OXTR | Cytokine_Receptors |
| PGR | Cytokine_Receptors |
| PGRMC2 | Cytokine_Receptors |
| PLAUR | Cytokine_Receptors |
| PLXNA1 | Cytokine_Receptors |
| PLXNA2 | Cytokine_Receptors |
| PLXNA3 | Cytokine_Receptors |
| PLXNA4 | Cytokine_Receptors |
| PLXNB1 | Cytokine_Receptors |
| PLXNB2 | Cytokine_Receptors |
| PLXNB3 | Cytokine_Receptors |
| PLXNC1 | Cytokine_Receptors |
| PLXND1 | Cytokine_Receptors |
| PPARA | Cytokine_Receptors |
| PPARD | Cytokine_Receptors |
| PPARG | Cytokine_Receptors |
| PRLHR | Cytokine_Receptors |
| PRLR | Cytokine_Receptors |
| PTAFR | Cytokine_Receptors |
| PTGDR | Cytokine_Receptors |
| PTGDS | Cytokine_Receptors |
| PTGER1 | Cytokine_Receptors |

| PTGER2 | Cytokine_Receptors |
| --- | --- |
| PTGER3 | Cytokine_Receptors |
| PTGER4 | Cytokine_Receptors |
| PTGFR | Cytokine_Receptors |
| PTH1R | Cytokine_Receptors |
| PTH2R | Cytokine_Receptors |
| RARA | Cytokine_Receptors |
| RARB | Cytokine_Receptors |
| RARG | Cytokine_Receptors |
| ROBO1 | Cytokine_Receptors |
| ROBO2 | Cytokine_Receptors |
| ROBO3 | Cytokine_Receptors |
| RORA | Cytokine_Receptors |
| RORB | Cytokine_Receptors |
| RORC | Cytokine_Receptors |
| RXFP1 | Cytokine_Receptors |
| RXFP2 | Cytokine_Receptors |
| RXFP3 | Cytokine_Receptors |
| RXRA | Cytokine_Receptors |
| RXRB | Cytokine_Receptors |
| RXRG | Cytokine_Receptors |
| S1PR1 | Cytokine_Receptors |
| S1PR2 | Cytokine_Receptors |
| SCTR | Cytokine_Receptors |
| SDC1 | Cytokine_Receptors |
| SDC2 | Cytokine_Receptors |
| SDC3 | Cytokine_Receptors |
| SDC4 | Cytokine_Receptors |
| SORT1 | Cytokine_Receptors |
| SSTR1 | Cytokine_Receptors |
| SSTR2 | Cytokine_Receptors |
| SSTR5 | Cytokine_Receptors |
| ST2 | Cytokine_Receptors |
| TACR1 | Cytokine_Receptors |
| TEK | Cytokine_Receptors |
| TGFBR1 | Cytokine_Receptors |
| TGFBR2 | Cytokine_Receptors |
| TGFBR3 | Cytokine_Receptors |
| THRA | Cytokine_Receptors |
| THRB | Cytokine_Receptors |
| TIE1 | Cytokine_Receptors |
| TNFRSF10A | Cytokine_Receptors |
| TNFRSF10B | Cytokine_Receptors |
| TNFRSF10C | Cytokine_Receptors |
| TNFRSF10D | Cytokine_Receptors |
| TNFRSF11A | Cytokine_Receptors |
| TNFRSF12A | Cytokine_Receptors |
| TNFRSF13B | Cytokine_Receptors |
| TNFRSF13C | Cytokine_Receptors |
| TNFRSF14 | Cytokine_Receptors |
| TNFRSF17 | Cytokine_Receptors |
| TNFRSF18 | Cytokine_Receptors |

| TNFRSF19 | Cytokine_Receptors |
| --- | --- |
| TNFRSF1A | Cytokine_Receptors |
| TNFRSF1B | Cytokine_Receptors |
| TNFRSF21 | Cytokine_Receptors |
| TNFRSF25 | Cytokine_Receptors |
| TNFRSF4 | Cytokine_Receptors |
| TNFRSF6B | Cytokine_Receptors |
| TNFRSF8 | Cytokine_Receptors |
| TNFRSF9 | Cytokine_Receptors |
| TRHR | Cytokine_Receptors |
| TSHR | Cytokine_Receptors |
| TUBB3 | Cytokine_Receptors |
| VDR | Cytokine_Receptors |
| VIPR1 | Cytokine_Receptors |
| VIPR2 | Cytokine_Receptors |
| XCR1 | Cytokine_Receptors |
| IFNA10 | Interferons |
| IFNA13 | Interferons |
| IFNA14 | Interferons |
| IFNA16 | Interferons |
| IFNA17 | Interferons |
| IFNA2 | Interferons |
| IFNA21 | Interferons |
| IFNA4 | Interferons |
| IFNA5 | Interferons |
| IFNA6 | Interferons |
| IFNA7 | Interferons |
| IFNA8 | Interferons |
| IFNB1 | Interferons |
| IFNE | Interferons |
| IFNG | Interferons |
| IFNK | Interferons |
| IFNW1 | Interferons |
| IFNAR2 | Interferon_Receptor |
| IFNGR1 | Interferon_Receptor |
| IFNGR2 | Interferon_Receptor |
| IL11 | Interleukins |
| IL12A | Interleukins |
| IL12B | Interleukins |
| IL13 | Interleukins |
| IL15 | Interleukins |
| IL16 | Interleukins |
| IL17A | Interleukins |
| IL17B | Interleukins |
| IL17C | Interleukins |
| IL17D | Interleukins |
| IL17F | Interleukins |
| IL18 | Interleukins |
| IL19 | Interleukins |
| IL1A | Interleukins |
| IL1B | Interleukins |
| IL1F10 | Interleukins |

| IL1F5 | Interleukins |
| --- | --- |
| IL1F6 | Interleukins |
| IL1F7 | Interleukins |
| IL1F8 | Interleukins |
| IL1F9 | Interleukins |
| IL1RN | Interleukins |
| IL2 | Interleukins |
| IL20 | Interleukins |
| IL21 | Interleukins |
| IL22 | Interleukins |
| IL23A | Interleukins |
| IL24 | Interleukins |
| IL25 | Interleukins |
| IL26 | Interleukins |
| IL27 | Interleukins |
| IL28A | Interleukins |
| IL28B | Interleukins |
| IL29 | Interleukins |
| IL3 | Interleukins |
| IL31 | Interleukins |
| IL32 | Interleukins |
| IL33 | Interleukins |
| IL34 | Interleukins |
| IL4 | Interleukins |
| IL5 | Interleukins |
| IL6 | Interleukins |
| IL6ST | Interleukins |
| IL7 | Interleukins |
| IL8 | Interleukins |
| IL9 | Interleukins |
| TXLNA | Interleukins |
| IL10RA | Interleukins_Receptor |
| IL10RB | Interleukins_Receptor |
| IL11RA | Interleukins_Receptor |
| IL11RB | Interleukins_Receptor |
| IL12RB1 | Interleukins_Receptor |
| IL12RB2 | Interleukins_Receptor |
| IL13RA1 | Interleukins_Receptor |
| IL13RA2 | Interleukins_Receptor |
| IL15RA | Interleukins_Receptor |
| IL15RB | Interleukins_Receptor |
| IL17RA | Interleukins_Receptor |
| IL17RB | Interleukins_Receptor |
| IL17RC | Interleukins_Receptor |
| IL17RD | Interleukins_Receptor |
| IL17RE | Interleukins_Receptor |
| IL18R1 | Interleukins_Receptor |
| IL18RAP | Interleukins_Receptor |
| IL1R1 | Interleukins_Receptor |
| IL1R2 | Interleukins_Receptor |
| IL1RAP | Interleukins_Receptor |
| IL1RL1 | Interleukins_Receptor |

| IL1RL2 | Interleukins_Receptor |
| --- | --- |
| IL20RA | Interleukins_Receptor |
| IL20RB | Interleukins_Receptor |
| IL21R | Interleukins_Receptor |
| IL22RA1 | Interleukins_Receptor |
| IL22RA2 | Interleukins_Receptor |
| IL23R | Interleukins_Receptor |
| IL27RA | Interleukins_Receptor |
| IL28RA | Interleukins_Receptor |
| IL2RA | Interleukins_Receptor |
| IL2RB | Interleukins_Receptor |
| IL2RG | Interleukins_Receptor |
| IL31RA | Interleukins_Receptor |
| IL3RA | Interleukins_Receptor |
| IL4R | Interleukins_Receptor |
| IL5RA | Interleukins_Receptor |
| IL6R | Interleukins_Receptor |
| IL7R | Interleukins_Receptor |
| IL8RA | Interleukins_Receptor |
| IL8RB | Interleukins_Receptor |
| IL9R | Interleukins_Receptor |
| ST2 | Interleukins_Receptor |
| HLA-A | NaturalKiller_Cell_Cytotoxicity |
| HLA-B | NaturalKiller_Cell_Cytotoxicity |
| HLA-C | NaturalKiller_Cell_Cytotoxicity |
| HLA-E | NaturalKiller_Cell_Cytotoxicity |
| HLA-G | NaturalKiller_Cell_Cytotoxicity |
| KIR3DL1 | NaturalKiller_Cell_Cytotoxicity |
| KIR3DL2 | NaturalKiller_Cell_Cytotoxicity |
| KIR2DL1 | NaturalKiller_Cell_Cytotoxicity |
| KIR2DL2 | NaturalKiller_Cell_Cytotoxicity |
| KIR2DL3 | NaturalKiller_Cell_Cytotoxicity |
| KIR2DL4 | NaturalKiller_Cell_Cytotoxicity |
| KIR2DL5A | NaturalKiller_Cell_Cytotoxicity |
| KLRC1 | NaturalKiller_Cell_Cytotoxicity |
| KLRC2 | NaturalKiller_Cell_Cytotoxicity |
| KLRC3 | NaturalKiller_Cell_Cytotoxicity |
| KLRD1 | NaturalKiller_Cell_Cytotoxicity |
| PTPN6 | NaturalKiller_Cell_Cytotoxicity |
| PTPN11 | NaturalKiller_Cell_Cytotoxicity |
| ICAM1 | NaturalKiller_Cell_Cytotoxicity |
| ICAM2 | NaturalKiller_Cell_Cytotoxicity |
| ITGAL | NaturalKiller_Cell_Cytotoxicity |
| ITGB2 | NaturalKiller_Cell_Cytotoxicity |
| PTK2B | NaturalKiller_Cell_Cytotoxicity |
| VAV3 | NaturalKiller_Cell_Cytotoxicity |
| VAV1 | NaturalKiller_Cell_Cytotoxicity |
| VAV2 | NaturalKiller_Cell_Cytotoxicity |
| RAC1 | NaturalKiller_Cell_Cytotoxicity |
| RAC2 | NaturalKiller_Cell_Cytotoxicity |
| RAC3 | NaturalKiller_Cell_Cytotoxicity |
| PAK1 | NaturalKiller_Cell_Cytotoxicity |

| MAP2K1 | NaturalKiller_Cell_Cytotoxicity |
| --- | --- |
| MAP2K2 | NaturalKiller_Cell_Cytotoxicity |
| MAPK1 | NaturalKiller_Cell_Cytotoxicity |
| MAPK3 | NaturalKiller_Cell_Cytotoxicity |
| TNF | NaturalKiller_Cell_Cytotoxicity |
| CSF2 | NaturalKiller_Cell_Cytotoxicity |
| IFNG | NaturalKiller_Cell_Cytotoxicity |
| KIR2DS1 | NaturalKiller_Cell_Cytotoxicity |
| KIR2DS3 | NaturalKiller_Cell_Cytotoxicity |
| KIR2DS4 | NaturalKiller_Cell_Cytotoxicity |
| KIR2DS5 | NaturalKiller_Cell_Cytotoxicity |
| NCR2 | NaturalKiller_Cell_Cytotoxicity |
| TYROBP | NaturalKiller_Cell_Cytotoxicity |
| LCK | NaturalKiller_Cell_Cytotoxicity |
| FCGR3A | NaturalKiller_Cell_Cytotoxicity |
| FCGR3B | NaturalKiller_Cell_Cytotoxicity |
| LOC652578 | NaturalKiller_Cell_Cytotoxicity |
| NCR1 | NaturalKiller_Cell_Cytotoxicity |
| NCR3 | NaturalKiller_Cell_Cytotoxicity |
| FCER1G | NaturalKiller_Cell_Cytotoxicity |
| CD247 | NaturalKiller_Cell_Cytotoxicity |
| ZAP70 | NaturalKiller_Cell_Cytotoxicity |
| SYK | NaturalKiller_Cell_Cytotoxicity |
| LCP2 | NaturalKiller_Cell_Cytotoxicity |
| LAT | NaturalKiller_Cell_Cytotoxicity |
| PLCG1 | NaturalKiller_Cell_Cytotoxicity |
| PLCG2 | NaturalKiller_Cell_Cytotoxicity |
| SH3BP2 | NaturalKiller_Cell_Cytotoxicity |
| PIK3CA | NaturalKiller_Cell_Cytotoxicity |
| PIK3CB | NaturalKiller_Cell_Cytotoxicity |
| PIK3CD | NaturalKiller_Cell_Cytotoxicity |
| PIK3CG | NaturalKiller_Cell_Cytotoxicity |
| PIK3R5 | NaturalKiller_Cell_Cytotoxicity |
| PIK3R1 | NaturalKiller_Cell_Cytotoxicity |
| PIK3R2 | NaturalKiller_Cell_Cytotoxicity |
| PIK3R3 | NaturalKiller_Cell_Cytotoxicity |
| FYN | NaturalKiller_Cell_Cytotoxicity |
| SHC2 | NaturalKiller_Cell_Cytotoxicity |
| SHC4 | NaturalKiller_Cell_Cytotoxicity |
| SHC3 | NaturalKiller_Cell_Cytotoxicity |
| SHC1 | NaturalKiller_Cell_Cytotoxicity |
| GRB2 | NaturalKiller_Cell_Cytotoxicity |
| SOS1 | NaturalKiller_Cell_Cytotoxicity |
| SOS2 | NaturalKiller_Cell_Cytotoxicity |
| HRAS | NaturalKiller_Cell_Cytotoxicity |
| KRAS | NaturalKiller_Cell_Cytotoxicity |
| NRAS | NaturalKiller_Cell_Cytotoxicity |
| ARAF | NaturalKiller_Cell_Cytotoxicity |
| BRAF | NaturalKiller_Cell_Cytotoxicity |
| RAF1 | NaturalKiller_Cell_Cytotoxicity |
| MICA | NaturalKiller_Cell_Cytotoxicity |
| MICB | NaturalKiller_Cell_Cytotoxicity |

| ULBP3 | NaturalKiller_Cell_Cytotoxicity |
| --- | --- |
| ULBP2 | NaturalKiller_Cell_Cytotoxicity |
| ULBP1 | NaturalKiller_Cell_Cytotoxicity |
| KLRK1 | NaturalKiller_Cell_Cytotoxicity |
| HCST | NaturalKiller_Cell_Cytotoxicity |
| CD48 | NaturalKiller_Cell_Cytotoxicity |
| CD244 | NaturalKiller_Cell_Cytotoxicity |
| PPP3CA | NaturalKiller_Cell_Cytotoxicity |
| PPP3CB | NaturalKiller_Cell_Cytotoxicity |
| PPP3CC | NaturalKiller_Cell_Cytotoxicity |
| CHP | NaturalKiller_Cell_Cytotoxicity |
| PPP3R1 | NaturalKiller_Cell_Cytotoxicity |
| PPP3R2 | NaturalKiller_Cell_Cytotoxicity |
| CHP2 | NaturalKiller_Cell_Cytotoxicity |
| NFAT5 | NaturalKiller_Cell_Cytotoxicity |
| NFATC1 | NaturalKiller_Cell_Cytotoxicity |
| NFATC2 | NaturalKiller_Cell_Cytotoxicity |
| NFATC3 | NaturalKiller_Cell_Cytotoxicity |
| NFATC4 | NaturalKiller_Cell_Cytotoxicity |
| PRKCA | NaturalKiller_Cell_Cytotoxicity |
| PRKCB | NaturalKiller_Cell_Cytotoxicity |
| PRKCG | NaturalKiller_Cell_Cytotoxicity |
| SH2D1B | NaturalKiller_Cell_Cytotoxicity |
| SH2D1A | NaturalKiller_Cell_Cytotoxicity |
| IFNGR1 | NaturalKiller_Cell_Cytotoxicity |
| IFNGR2 | NaturalKiller_Cell_Cytotoxicity |
| IFNA1 | NaturalKiller_Cell_Cytotoxicity |
| IFNA2 | NaturalKiller_Cell_Cytotoxicity |
| IFNA4 | NaturalKiller_Cell_Cytotoxicity |
| IFNA5 | NaturalKiller_Cell_Cytotoxicity |
| IFNA6 | NaturalKiller_Cell_Cytotoxicity |
| IFNA7 | NaturalKiller_Cell_Cytotoxicity |
| IFNA8 | NaturalKiller_Cell_Cytotoxicity |
| IFNA10 | NaturalKiller_Cell_Cytotoxicity |
| IFNA13 | NaturalKiller_Cell_Cytotoxicity |
| IFNA14 | NaturalKiller_Cell_Cytotoxicity |
| IFNA16 | NaturalKiller_Cell_Cytotoxicity |
| IFNA17 | NaturalKiller_Cell_Cytotoxicity |
| IFNA21 | NaturalKiller_Cell_Cytotoxicity |
| IFNB1 | NaturalKiller_Cell_Cytotoxicity |
| IFNAR1 | NaturalKiller_Cell_Cytotoxicity |
| IFNAR2 | NaturalKiller_Cell_Cytotoxicity |
| TNFSF10 | NaturalKiller_Cell_Cytotoxicity |
| TNFRSF10D | NaturalKiller_Cell_Cytotoxicity |
| TNFRSF10C | NaturalKiller_Cell_Cytotoxicity |
| TNFRSF10B | NaturalKiller_Cell_Cytotoxicity |
| TNFRSF10A | NaturalKiller_Cell_Cytotoxicity |
| FASLG | NaturalKiller_Cell_Cytotoxicity |
| FAS | NaturalKiller_Cell_Cytotoxicity |
| GZMB | NaturalKiller_Cell_Cytotoxicity |
| PRF1 | NaturalKiller_Cell_Cytotoxicity |
| CASP3 | NaturalKiller_Cell_Cytotoxicity |

| BID | NaturalKiller_Cell_Cytotoxicity |
| --- | --- |
| CD3D | TCRsignalingPathway |
| CD3E | TCRsignalingPathway |
| CD3G | TCRsignalingPathway |
| CD247 | TCRsignalingPathway |
| CD4 | TCRsignalingPathway |
| CD8A | TCRsignalingPathway |
| CD8B | TCRsignalingPathway |
| PTPRC | TCRsignalingPathway |
| LCK | TCRsignalingPathway |
| FYN | TCRsignalingPathway |
| ZAP70 | TCRsignalingPathway |
| LCP2 | TCRsignalingPathway |
| LAT | TCRsignalingPathway |
| ITK | TCRsignalingPathway |
| TEC | TCRsignalingPathway |
| NCK1 | TCRsignalingPathway |
| NCK2 | TCRsignalingPathway |
| VAV3 | TCRsignalingPathway |
| VAV1 | TCRsignalingPathway |
| VAV2 | TCRsignalingPathway |
| GRAP2 | TCRsignalingPathway |
| GRB2 | TCRsignalingPathway |
| PAK1 | TCRsignalingPathway |
| PAK2 | TCRsignalingPathway |
| PAK3 | TCRsignalingPathway |
| PAK4 | TCRsignalingPathway |
| PAK6 | TCRsignalingPathway |
| PAK7 | TCRsignalingPathway |
| RHOA | TCRsignalingPathway |
| CDC42 | TCRsignalingPathway |
| PPP3CA | TCRsignalingPathway |
| PPP3CB | TCRsignalingPathway |
| PPP3CC | TCRsignalingPathway |
| CHP | TCRsignalingPathway |
| PPP3R1 | TCRsignalingPathway |
| PPP3R2 | TCRsignalingPathway |
| CHP2 | TCRsignalingPathway |
| NFAT5 | TCRsignalingPathway |
| NFATC1 | TCRsignalingPathway |
| NFATC2 | TCRsignalingPathway |
| NFATC3 | TCRsignalingPathway |
| NFATC4 | TCRsignalingPathway |
| SOS1 | TCRsignalingPathway |
| SOS2 | TCRsignalingPathway |
| HRAS | TCRsignalingPathway |
| KRAS | TCRsignalingPathway |
| NRAS | TCRsignalingPathway |
| FOS | TCRsignalingPathway |
| JUN | TCRsignalingPathway |
| CARD11 | TCRsignalingPathway |
| BCL10 | TCRsignalingPathway |

| MALT1 | TCRsignalingPathway |
| --- | --- |
| CHUK | TCRsignalingPathway |
| IKBKB | TCRsignalingPathway |
| IKBKG | TCRsignalingPathway |
| NFKB1 | TCRsignalingPathway |
| RELA | TCRsignalingPathway |
| NFKBIA | TCRsignalingPathway |
| NFKBIB | TCRsignalingPathway |
| NFKBIE | TCRsignalingPathway |
| CD28 | TCRsignalingPathway |
| ICOS | TCRsignalingPathway |
| CD40LG | TCRsignalingPathway |
| PIK3R5 | TCRsignalingPathway |
| PIK3R1 | TCRsignalingPathway |
| PIK3R2 | TCRsignalingPathway |
| PIK3R3 | TCRsignalingPathway |
| PIK3CA | TCRsignalingPathway |
| PIK3CB | TCRsignalingPathway |
| PIK3CD | TCRsignalingPathway |
| PIK3CG | TCRsignalingPathway |
| AKT3 | TCRsignalingPathway |
| AKT1 | TCRsignalingPathway |
| AKT2 | TCRsignalingPathway |
| MAP3K8 | TCRsignalingPathway |
| MAP3K14 | TCRsignalingPathway |
| PDCD1 | TCRsignalingPathway |
| CTLA4 | TCRsignalingPathway |
| PTPN6 | TCRsignalingPathway |
| CBLC | TCRsignalingPathway |
| CBL | TCRsignalingPathway |
| CBLB | TCRsignalingPathway |
| IL2 | TCRsignalingPathway |
| IL4 | TCRsignalingPathway |
| IL5 | TCRsignalingPathway |
| IL10 | TCRsignalingPathway |
| IFNG | TCRsignalingPathway |
| CSF2 | TCRsignalingPathway |
| TNF | TCRsignalingPathway |
| CDK4 | TCRsignalingPathway |
| RASGRP1 | TCRsignalingPathway |
| PDK1 | TCRsignalingPathway |
| PLCG1 | TCRsignalingPathway |
| PRKCQ | TCRsignalingPathway |
| TRAC | TCRsignalingPathway |
| TRAJ1 | TCRsignalingPathway |
| TRAJ2 | TCRsignalingPathway |
| TRAJ3 | TCRsignalingPathway |
| TRAJ4 | TCRsignalingPathway |
| TRAJ5 | TCRsignalingPathway |
| TRAJ6 | TCRsignalingPathway |
| TRAJ7 | TCRsignalingPathway |
| TRAJ8 | TCRsignalingPathway |

| TRAJ9 | TCRsignalingPathway |
| --- | --- |
| TRAJ10 | TCRsignalingPathway |
| TRAJ11 | TCRsignalingPathway |
| TRAJ12 | TCRsignalingPathway |
| TRAJ13 | TCRsignalingPathway |
| TRAJ14 | TCRsignalingPathway |
| TRAJ15 | TCRsignalingPathway |
| TRAJ16 | TCRsignalingPathway |
| TRAJ17 | TCRsignalingPathway |
| TRAJ18 | TCRsignalingPathway |
| TRAJ19 | TCRsignalingPathway |
| TRAJ20 | TCRsignalingPathway |
| TRAJ21 | TCRsignalingPathway |
| TRAJ22 | TCRsignalingPathway |
| TRAJ23 | TCRsignalingPathway |
| TRAJ24 | TCRsignalingPathway |
| TRAJ25 | TCRsignalingPathway |
| TRAJ26 | TCRsignalingPathway |
| TRAJ27 | TCRsignalingPathway |
| TRAJ28 | TCRsignalingPathway |
| TRAJ29 | TCRsignalingPathway |
| TRAJ30 | TCRsignalingPathway |
| TRAJ31 | TCRsignalingPathway |
| TRAJ32 | TCRsignalingPathway |
| TRAJ33 | TCRsignalingPathway |
| TRAJ34 | TCRsignalingPathway |
| TRAJ35 | TCRsignalingPathway |
| TRAJ36 | TCRsignalingPathway |
| TRAJ37 | TCRsignalingPathway |
| TRAJ38 | TCRsignalingPathway |
| TRAJ39 | TCRsignalingPathway |
| TRAJ40 | TCRsignalingPathway |
| TRAJ41 | TCRsignalingPathway |
| TRAJ42 | TCRsignalingPathway |
| TRAJ43 | TCRsignalingPathway |
| TRAJ44 | TCRsignalingPathway |
| TRAJ45 | TCRsignalingPathway |
| TRAJ46 | TCRsignalingPathway |
| TRAJ47 | TCRsignalingPathway |
| TRAJ48 | TCRsignalingPathway |
| TRAJ49 | TCRsignalingPathway |
| TRAJ50 | TCRsignalingPathway |
| TRAJ52 | TCRsignalingPathway |
| TRAJ53 | TCRsignalingPathway |
| TRAJ54 | TCRsignalingPathway |
| TRAJ56 | TCRsignalingPathway |
| TRAJ57 | TCRsignalingPathway |
| TRAJ58 | TCRsignalingPathway |
| TRAJ59 | TCRsignalingPathway |
| TRAJ61 | TCRsignalingPathway |
| TRAV1-1 | TCRsignalingPathway |
| TRAV1-2 | TCRsignalingPathway |

| TRAV2 | TCRsignalingPathway |
| --- | --- |
| TRAV3 | TCRsignalingPathway |
| TRAV4 | TCRsignalingPathway |
| TRAV5 | TCRsignalingPathway |
| TRAV7 | TCRsignalingPathway |
| TRAV8-1 | TCRsignalingPathway |
| TRAV8-2 | TCRsignalingPathway |
| TRAV8-3 | TCRsignalingPathway |
| TRAV8-4 | TCRsignalingPathway |
| TRAV8-6 | TCRsignalingPathway |
| TRAV8-7 | TCRsignalingPathway |
| TRAV9-1 | TCRsignalingPathway |
| TRAV9-2 | TCRsignalingPathway |
| TRAV10 | TCRsignalingPathway |
| TRAV12-1 | TCRsignalingPathway |
| TRAV12-2 | TCRsignalingPathway |
| TRAV12-3 | TCRsignalingPathway |
| TRAV13-1 | TCRsignalingPathway |
| TRAV13-2 | TCRsignalingPathway |
| TRAV14DV4 | TCRsignalingPathway |
| TRAV16 | TCRsignalingPathway |
| TRAV17 | TCRsignalingPathway |
| TRAV18 | TCRsignalingPathway |
| TRAV19 | TCRsignalingPathway |
| TRAV20 | TCRsignalingPathway |
| TRAV21 | TCRsignalingPathway |
| TRAV22 | TCRsignalingPathway |
| TRAV23DV6 | TCRsignalingPathway |
| TRAV24 | TCRsignalingPathway |
| TRAV25 | TCRsignalingPathway |
| TRAV26-1 | TCRsignalingPathway |
| TRAV26-2 | TCRsignalingPathway |
| TRAV27 | TCRsignalingPathway |
| TRAV29DV5 | TCRsignalingPathway |
| TRAV30 | TCRsignalingPathway |
| TRAV34 | TCRsignalingPathway |
| TRAV35 | TCRsignalingPathway |
| TRAV36DV7 | TCRsignalingPathway |
| TRAV38-1 | TCRsignalingPathway |
| TRAV38-2DV8 | TCRsignalingPathway |
| TRAV39 | TCRsignalingPathway |
| TRAV40 | TCRsignalingPathway |
| TRAV41 | TCRsignalingPathway |
| TRBC1 | TCRsignalingPathway |
| TRBC2 | TCRsignalingPathway |
| TRBD1 | TCRsignalingPathway |
| TRBD2 | TCRsignalingPathway |
| TRBJ1-1 | TCRsignalingPathway |
| TRBJ1-2 | TCRsignalingPathway |
| TRBJ1-3 | TCRsignalingPathway |
| TRBJ1-4 | TCRsignalingPathway |
| TRBJ1-5 | TCRsignalingPathway |

| TRBJ1-6 | TCRsignalingPathway |
| --- | --- |
| TRBJ2-1 | TCRsignalingPathway |
| TRBJ2-2 | TCRsignalingPathway |
| TRBJ2-3 | TCRsignalingPathway |
| TRBJ2-4 | TCRsignalingPathway |
| TRBJ2-5 | TCRsignalingPathway |
| TRBJ2-6 | TCRsignalingPathway |
| TRBJ2-7 | TCRsignalingPathway |
| TRBV2 | TCRsignalingPathway |
| TRBV3-1 | TCRsignalingPathway |
| TRBV4-1 | TCRsignalingPathway |
| TRBV4-2 | TCRsignalingPathway |
| TRBV4-3 | TCRsignalingPathway |
| TRBV5-1 | TCRsignalingPathway |
| TRBV5-4 | TCRsignalingPathway |
| TRBV5-5 | TCRsignalingPathway |
| TRBV5-6 | TCRsignalingPathway |
| TRBV5-7 | TCRsignalingPathway |
| TRBV5-8 | TCRsignalingPathway |
| TRBV6-1 | TCRsignalingPathway |
| TRBV6-2 | TCRsignalingPathway |
| TRBV6-3 | TCRsignalingPathway |
| TRBV6-4 | TCRsignalingPathway |
| TRBV6-5 | TCRsignalingPathway |
| TRBV6-6 | TCRsignalingPathway |
| TRBV6-7 | TCRsignalingPathway |
| TRBV6-8 | TCRsignalingPathway |
| TRBV6-9 | TCRsignalingPathway |
| TRBV7-2 | TCRsignalingPathway |
| TRBV7-3 | TCRsignalingPathway |
| TRBV7-4 | TCRsignalingPathway |
| TRBV7-6 | TCRsignalingPathway |
| TRBV7-7 | TCRsignalingPathway |
| TRBV7-8 | TCRsignalingPathway |
| TRBV7-9 | TCRsignalingPathway |
| TRBV9 | TCRsignalingPathway |
| TRBV10-1 | TCRsignalingPathway |
| TRBV10-2 | TCRsignalingPathway |
| TRBV10-3 | TCRsignalingPathway |
| TRBV11-1 | TCRsignalingPathway |
| TRBV11-2 | TCRsignalingPathway |
| TRBV11-3 | TCRsignalingPathway |
| TRBV12-3 | TCRsignalingPathway |
| TRBV12-4 | TCRsignalingPathway |
| TRBV12-5 | TCRsignalingPathway |
| TRBV13 | TCRsignalingPathway |
| TRBV14 | TCRsignalingPathway |
| TRBV15 | TCRsignalingPathway |
| TRBV16 | TCRsignalingPathway |
| TRBV17 | TCRsignalingPathway |
| TRBV18 | TCRsignalingPathway |
| TRBV19 | TCRsignalingPathway |

| TRBV20-1 | TCRsignalingPathway |
| --- | --- |
| TRBV24-1 | TCRsignalingPathway |
| TRBV25-1 | TCRsignalingPathway |
| TRBV27 | TCRsignalingPathway |
| TRBV28 | TCRsignalingPathway |
| TRBV29-1 | TCRsignalingPathway |
| TRBV30 | TCRsignalingPathway |
| TRDC | TCRsignalingPathway |
| TRDD1 | TCRsignalingPathway |
| TRDD2 | TCRsignalingPathway |
| TRDD3 | TCRsignalingPathway |
| TRDJ1 | TCRsignalingPathway |
| TRDJ2 | TCRsignalingPathway |
| TRDJ3 | TCRsignalingPathway |
| TRDJ4 | TCRsignalingPathway |
| TRDV1 | TCRsignalingPathway |
| TRDV2 | TCRsignalingPathway |
| TRDV3 | TCRsignalingPathway |
| TRGV9 | TCRsignalingPathway |
| TRGV8 | TCRsignalingPathway |
| TRGV5 | TCRsignalingPathway |
| TRGV4 | TCRsignalingPathway |
| TRGV3 | TCRsignalingPathway |
| TRGV2 | TCRsignalingPathway |
| TRGJP2 | TCRsignalingPathway |
| TRGJP1 | TCRsignalingPathway |
| TRGJP | TCRsignalingPathway |
| TRGJ2 | TCRsignalingPathway |
| TRGJ1 | TCRsignalingPathway |
| TRGC2 | TCRsignalingPathway |
| TRGC1 | TCRsignalingPathway |
| TRAV6 | TCRsignalingPathway |
| BMP1 | TGFb_Family_Member |
| BMP10 | TGFb_Family_Member |
| BMP15 | TGFb_Family_Member |
| BMP2 | TGFb_Family_Member |
| BMP3 | TGFb_Family_Member |
| BMP4 | TGFb_Family_Member |
| BMP5 | TGFb_Family_Member |
| BMP6 | TGFb_Family_Member |
| BMP7 | TGFb_Family_Member |
| BMP8A | TGFb_Family_Member |
| BMP8B | TGFb_Family_Member |
| GDF1 | TGFb_Family_Member |
| GDF10 | TGFb_Family_Member |
| GDF11 | TGFb_Family_Member |
| GDF15 | TGFb_Family_Member |
| GDF2 | TGFb_Family_Member |
| GDF3 | TGFb_Family_Member |
| GDF5 | TGFb_Family_Member |
| GDF6 | TGFb_Family_Member |
| GDF7 | TGFb_Family_Member |

| GDF9 | TGFb_Family_Member |
| --- | --- |
| GDNF | TGFb_Family_Member |
| INHA | TGFb_Family_Member |
| INHBA | TGFb_Family_Member |
| INHBB | TGFb_Family_Member |
| INHBC | TGFb_Family_Member |
| INHBE | TGFb_Family_Member |
| LEFTY1 | TGFb_Family_Member |
| LEFTY2 | TGFb_Family_Member |
| NODAL | TGFb_Family_Member |
| TGFB1 | TGFb_Family_Member |
| TGFB2 | TGFb_Family_Member |
| TGFB3 | TGFb_Family_Member |
| ACVR1B | TGFb_Family_Member_Receptor |
| ACVR1C | TGFb_Family_Member_Receptor |
| ACVR2A | TGFb_Family_Member_Receptor |
| ACVR2B | TGFb_Family_Member_Receptor |
| ACVRL1 | TGFb_Family_Member_Receptor |
| AMHR2 | TGFb_Family_Member_Receptor |
| BMPR1A | TGFb_Family_Member_Receptor |
| BMPR1B | TGFb_Family_Member_Receptor |
| BMPR2 | TGFb_Family_Member_Receptor |
| TGFBR1 | TGFb_Family_Member_Receptor |
| TGFBR2 | TGFb_Family_Member_Receptor |
| TGFBR3 | TGFb_Family_Member_Receptor |
| TNFRSF11B | TNF_Family_Members |
| TNFSF10 | TNF_Family_Members |
| TNFSF11 | TNF_Family_Members |
| TNFSF12 | TNF_Family_Members |
| TNFSF13 | TNF_Family_Members |
| TNFSF13B | TNF_Family_Members |
| TNFSF14 | TNF_Family_Members |
| TNFSF15 | TNF_Family_Members |
| TNFSF18 | TNF_Family_Members |
| TNFSF4 | TNF_Family_Members |
| TNFSF8 | TNF_Family_Members |
| TNFSF9 | TNF_Family_Members |
| TNFRSF10B | TNF_Family_Members_Receptors |
| TNFRSF10C | TNF_Family_Members_Receptors |
| TNFRSF10D | TNF_Family_Members_Receptors |
| TNFRSF11A | TNF_Family_Members_Receptors |
| TNFRSF12A | TNF_Family_Members_Receptors |
| TNFRSF13B | TNF_Family_Members_Receptors |
| TNFRSF13C | TNF_Family_Members_Receptors |
| TNFRSF14 | TNF_Family_Members_Receptors |
| TNFRSF17 | TNF_Family_Members_Receptors |
| TNFRSF18 | TNF_Family_Members_Receptors |
| TNFRSF19 | TNF_Family_Members_Receptors |
| TNFRSF1A | TNF_Family_Members_Receptors |
| TNFRSF1B | TNF_Family_Members_Receptors |
| TNFRSF21 | TNF_Family_Members_Receptors |
| TNFRSF25 | TNF_Family_Members_Receptors |

| TNFRSF4 | TNF_Family_Members_Receptors |
| --- | --- |
| TNFRSF6B | TNF_Family_Members_Receptors |
| TNFRSF8 | TNF_Family_Members_Receptors |
| TNFRSF9 | TNF_Family_Members_Receptors |
