# Supplementary material for: Single-cell and spatial dissection of necroptosis spatiotemporal evolution driving lymph node metastasis in gastric cancer
Source: Cell Death Discov. 2025 Nov 17;11:535. doi: 10.1038/s41420-025-02815-z (PMC12623802; doi:10.1038/s41420-025-02815-z)
Supplement: Supplementary file 4 — Support Table 2 [file 41420_2025_2815_MOESM4_ESM.docx]

| **Characteristics** | **Total (N)** | **OR (95% CI)** | **P value** |
| --- | --- | --- | --- |
| **Pathologic T stage (T3&T4 vs. T1&T2)** | **367** | **1.152 (0.726 – 1.829)** | **0.548** |
| **Pathologic N stage (N1&N2&N3 vs. N0)** | **357** | **1.934 (1.223 – 3.058)** | **0.005** |
| **Pathologic M stage (M1 vs. M0)** | **355** | **0.912 (0.404 – 2.058)** | **0.824** |
| **Gender (Male vs. Female)** | **375** | **1.609 (1.051 – 2.463)** | **0.029** |
| **Age (> 65 vs. <= 65)** | **371** | **0.907 (0.602 – 1.367)** | **0.642** |

**Table 2.Results of single - gene Logistic regression analysis.**
